# IgG and Fcγ Receptors in Intestinal Immunity and Inflammation

**DOI:** 10.3389/fimmu.2019.00805

**Published:** 2019-04-12

**Authors:** Tomas Castro-Dopico, Menna R. Clatworthy

**Affiliations:** ^1^Molecular Immunity Unit, MRC Laboratory of Molecular Biology, Department of Medicine, University of Cambridge, Cambridge, United Kingdom; ^2^NIHR Cambridge Biomedical Research Centre Cambridge, United Kingdom; ^3^Cellular Genetics, Wellcome Sanger Institute, Hinxton, United Kingdom

**Keywords:** Fcγ receptor, IgG, inflammatory bowel disease, intestinal immunity, mucosal infections, neonatal immunity

## Abstract

Fcγ receptors (FcγR) are cell surface glycoproteins that mediate cellular effector functions of immunoglobulin G (IgG) antibodies. Genetic variation in FcγR genes can influence susceptibility to a variety of antibody-mediated autoimmune and inflammatory disorders, including systemic lupus erythematosus (SLE) and rheumatoid arthritis (RA). More recently, however, genetic studies have implicated altered FcγR signaling in the pathogenesis of inflammatory bowel disease (IBD), a condition classically associated with dysregulated innate and T cell immunity. Specifically, a variant of the activating receptor, FcγRIIA, with low affinity for IgG, confers protection against the development of ulcerative colitis, a subset of IBD, leading to a re-evaluation of the role of IgG and FcγRs in gastrointestinal tract immunity, an organ system traditionally associated with IgA. In this review, we summarize our current understanding of IgG and FcγR function at this unique host-environment interface, from the pathogenesis of colitis and defense against enteropathogens, its contribution to maternal-fetal cross-talk and susceptibility to cancer. Finally, we discuss the therapeutic implications of this information, both in terms of how FcγR signaling pathways may be targeted for the treatment of IBD and how FcγR engagement may influence the efficacy of therapeutic monoclonal antibodies in IBD.

## Introduction

The gastrointestinal (GI) tract comprises a series of organs whose primary functions are digestion, absorption, excretion, and to host a vast and diverse community of microbial commensals. The stomach and small intestine contribute to physical and chemical digestion and absorption, while the colon is primarily involved in the desiccation and compaction of waste (1). To aid these functions, the human GI tract is colonized by trillions of microorganisms that together form the microbiome, including at least 1,000 species of bacteria, the major component of the commensal flora. Microbial colonization increases progressively along the GI tract, with the colon harboring 10^10^-10^12^ organisms per gram of luminal contents and elevated species diversity. In the lower GI tract of healthy individuals, anaerobes dominate, including *Bacteroides*, bifidobacterial, fusobacteria, and peptostreptococci, while aerobes, such as enterobacteria, are present at lower densities (2).

A state of mutualism exists between the host and the commensal microbiota, whereby bacteria benefit from the energy-rich sources of food, and the host salvages essential compounds from indigestible nutrients, such as dietary polysaccharides. In this scenario, the host immune system has an essential role in maintaining on-going symbiosis by limiting tissue invasion by resident microbes and keeping detrimental inflammatory responses at bay. This is achieved by dynamic cross-talk between microbes, intestinal epithelial cells (IECs), and tissue-resident leukocytes (3, 4) and includes the production of anti-microbial peptides and mucus by IECs, the induction and secretion of immunoglobulin (Ig)A by intestinal plasma cells, and the dominance of an anti-inflammatory milieu that suppresses damaging responses to luminal microbes. In return, the microbiota interacts with and educates the intestinal immune system, with consequences for both local and system inflammation.

While classically associated with systemic pro-inflammatory responses, recent studies have demonstrated that constitutive production of GI anti-microbial IgG is a feature of adult homeostasis with roles in immune cell maturation, defense against infection, and maternally-acquired neonatal immunity (5–8). Furthermore, historical observations of *de novo* anti-microbial and autoreactive IgG in patients with inflammatory bowel disease (IBD) (9–11) have been brought into renewed focus by the identification of a polymorphism in the activating receptor FcγRIIA that alters susceptibility to ulcerative colitis (UC) (12–14), with subsequent studies demonstrating the pathogenic role of anti-microbial IgG in colitis. In this review, we will address the role that IgG and subsequent Fcγ receptor (FcγR) engagement by local GI-resident immune cells plays in intestinal immunity and inflammation, and the consequence of this interaction for defense against infection, immune maturation, detrimental inflammatory disease, and cancer.

## IgG Subclasses and Effector Function

IgG antibodies are the most abundant immunoglobulin isotype in human serum and extracellular tissue fluid, accounting for 10–20% of all plasma protein and 70–75% of total Ig (15). IgG subclasses exhibit diverse effector functions, including the ability to activate complement via the binding and activation of C1q, the engagement of FcγRs on immune cells, and the direct neutralization of toxins and microbes (16). With pleiotropic roles in immunity, detrimental IgG-driven immune responses are associated with several inflammatory and autoimmune disorders, including systemic lupus erythematosus (SLE) and rheumatoid arthritis (RA) (17, 18), but IgG antibodies are also key effector molecules that contribute to anti-microbial immunity. Generally, IgG antibodies are known for their high antigen affinity, driven by somatic hypermutation, and are key molecules involved in immunological memory, although these functions vary depending on IgG subclass.

**Figure 1:**
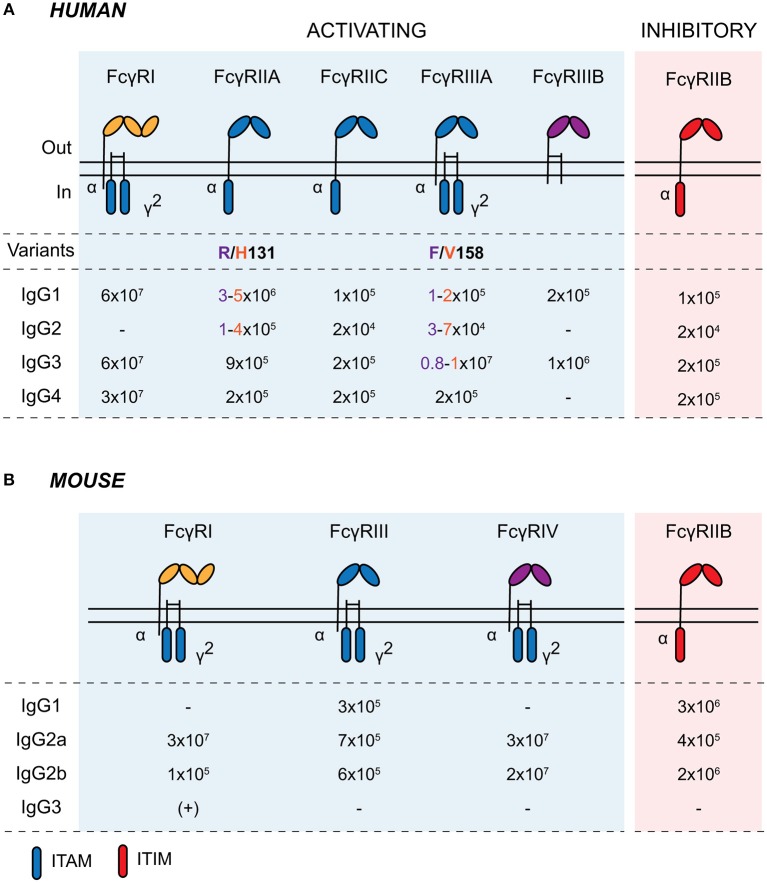
Human and murine Fcγ receptors. Schematic of human **(A)** and murine **(B)** classical Fcγ receptors embedded in the plasma membrane. Activating receptors contain intracellular ITAMs on the intracellular domain of the α chain or in the associated common γ-chain (γ^2^; encoded by *FCER1G*). Activating FcγRs are shaded in blue and inhibitory FcγRIIB is shaded in red. IgG affinity-altering variants are highlighted beneath the respective human FcγR, with the low- and high-binding variants and associated IgG affinities colored in purple and orange, respectively, in the table. Binding affinities are indicated as *K*_*A*_ (M^−1^). ITAM, immunoreceptor tyrosine-based activating motif; ITIM, immunoreceptor tyrosine-based inhibitory motif.

FcγRs are cell surface glycoproteins that bind to the Fc portion of IgG antibodies (19). They are widely expressed across cells of the immune system and are responsible for mediating the cellular effector functions of IgG, including immune cell migration and maturation, the production of inflammatory mediators, and the elimination of opsonized microbes (20). There are several activating FcγRs (FcγRI, FcγRIIA, FcγRIIIA, and FcγRIIIB in humans; FcγRI, FcγRIII, and FcγRIV in mice) and a single inhibitory receptor, FcγRIIB, in both humans and mice, with most exhibiting low-to-medium affinity for IgG (Figure 1). The neonatal Fc receptor (FcRn) and the intracellular tripartite motif-containing protein 21 (TRIM21) also bind to immunoglobulins following their internalization (15, 21). FcRn is a major histocompatibility complex (MHC) class I-like molecule that binds to the Fc domain of IgG in a 2:1 stoichiometry with micro- to nanomolar affinity at pH 6.5 within acidic endosomes (22). As well as protecting IgG from degradation with endothelial and myeloid cells, FcRn plays a key role in the active bidirectional transport of IgG across barrier surfaces. It is expressed by murine IECs until weaning and throughout life in the human GI tract. This allows the retrieval of IgG and IgG-antigen immune complexes from the gastrointestinal lumen for phagocytes within the lamina propria, as well as mediating the secretion of IgG (23–26).

**Figure 2:**
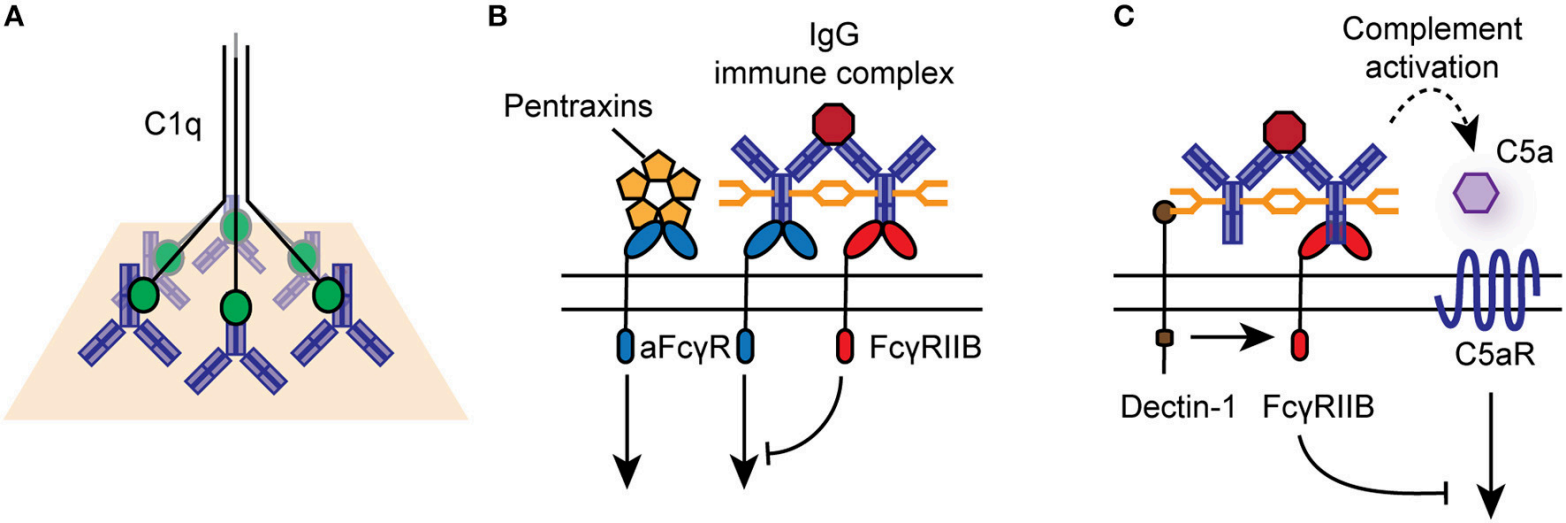
Effector functions of IgG antibodies and FcγRs. **(A)** IgG-opsonized antigens and cells can engage the classical complement pathway via binding to C1q. **(B)** IgG immune complexes, aggregated, or deposited IgG can bind to activating or inhibitory FcγRs on the surface of immune cells, particularly those of the innate immune system. Activating FcγRs (aFcγR) can also bind to pentraxins, such as CRP, linking to cellular responses to innate humoral immunity. **(C)** Specific glycosyl variants of IgG can bind to C-type lectin receptors, such as Dectin-1 and SIGN-R1, to mediated activating FcγR-independent immune function.

For classical FcγRs on the cell surface, productive signaling is initiated by the cross-linking of several receptors into signaling synapses on the cell surface through high-avidity antigen:antibody immune complexes (IC), aggregated IgG, or IgG-opsonized cells and surfaces (Figure 2). Upon cross-linking, phosphorylation of immunoreceptor tyrosine-based activating motifs (ITAMs) located on the intracellular domain of activating FcγRs or on the associated common γ-chain (also known as FcϵRIγ/FcRγ) occurs. ITAM phosphorylation activates signaling cascades via SRC family kinases and spleen tyrosine kinase (SYK), resulting in downstream activation of phosphatidylinositol3-kinase (PI3K) and phospholipase-Cγ. FcγRIIB contains an intracellular immunoreceptor tyrosine-based inhibitory motif (ITIM), which becomes phosphorylated upon cross-linking with activating FcγRs or the B cell receptor, initiating the recruitment of inositol phosphatases, most notably SHIP1, to the signaling synapse to dampen IgG-mediated responses (18). Activating and inhibitory FcγRs are co-expressed on many immune cells, and their relative expression and activity determines the activation threshold of cells upon encounter of ICs or opsonized targets.

While IgG is the dominant ligand for FcγRs, they can also signal in response to binding to other soluble effectors, such as pentraxins, including C reactive protein (CRP) and serum amyloid P (Figure 2) (27, 28). In particular, CRP mediates FcγRIIA-dependent internalization of damaged cells, self-antigens and microbes by human monocytes and neutrophils, via binding to phosphorylcholine moieties, chromatin, and histones (29, 30).

There are four IgG subclasses in humans (IgG1-4) and mice (IgG1, IgG2a/c, IgG2b, and IgG3). Human IgG1 is the most abundant and predominantly targets soluble protein antigens and membrane proteins (15). The generation of IgG1 is largely T cell dependent and it exhibits potent effector function (31). As such, IgG1 is frequently exploited in pharmaceutical settings for the production of therapeutic monoclonal antibodies (32). In mice, the effector profile of IgG2a and IgG2b is most similar to human IgG1 and show the greatest effector function *in vivo* in several settings (17). Human IgG2 responses (IgG3 in mice) are almost completely restricted to T cell-independent bacterial capsular carbohydrates, although anti-carbohydrate IgG antibodies of other subclasses do exist (33). IgG2 and IgG4 antibodies have a short, rigid hinge region compared to IgG1 and 3, resulting in impaired antibody flexibility, and this dictates to a certain extent the affinity of these molecules for FcγRs and C1q. Human IgG3 antibodies are the most effective subclass in terms of their activating effector functions, with enhanced binding to C1q and increased affinity for FcγRs, but they exhibit a blunted half-life due to impaired recycling via the FcRn (34). Finally, IgG4 is associated with induction by allergens following repeated or long-term exposure to antigen in a non-infectious setting, as well as in immune responses to parasitic infections (15). Given its relatively high affinity for the inhibitory receptor FcγRIIB, an ability to spontaneously dissociate and form bispecific antibodies (35), and the capacity to compete with IgE for allergens, IgG4 is often seen as an inhibitor of effector responses (36).

Beyond subclass, IgG effector functions can be fine-tuned through post-translational modification, most notably via *N-*linked glycosylation, which alters antibody stability, FcγR affinity and complement activity (37–40). Each IgG heavy chain carries a single covalently attached biantennary *N-*glycan at the highly conserved asparagine 297 residue in each of the Fc fragment Cγ2 domains, with over 900 IgG glycoforms possible (41). Biantennary complexes can contain additional bisecting *N-*acetylglucosamine (GlcNAc), core fucose, galactose and sialic acid residues (42).

Defucosylated IgG exhibits enhanced FcγRIIIA affinity (43), while sialylation promotes anti-inflammatory functions of IgG by reducing FcγR affinity and promoting binding to the C-type lectin receptor, SIGN-R1 (DC-SIGN in humans) (37, 44, 45). Indeed, sialylation is required for some of the protective functions of intravenous immunoglobulin (IVIg). Agalactosylated IgG, with two oligosaccharide chains ending in GlcNAc rather than galactose/sialic acid, is termed G0 (no galactose) (19). G0 IgG can activate complement via binding to mannose binding lectin and can bind the mannose receptor on phagocytes (46, 47).

In summary IgG antibodies are powerful effector molecules that can mediate tissue inflammation by complement activation, engagement of classical FcγRs and C-type lectin receptors (Figure 2). They are the dominant circulating antibody in humans and mice, with documented roles in defense and autoimmunity but their contribution to immunity in the gastrointestinal tract is much less well-defined.

## Immunity in the Gastrointestinal Tract

The frontline barrier between the external environment in the lumen of the GI tract and host tissues is the intestinal epithelium. This physical and biochemical barrier consists of an outer-most layer of thick secreted mucus, a single-cell layer of IECs, and the underlying non-epithelial mucosal cells, including a network of leukocytes found within the lamina propria, muscularis layers, and organized lymphoid follicles (48).

The largest mucosal barrier in the human body, the intestinal epithelium is continuously renewed by pluripotent intestinal epithelial stem cells located in the base of the crypts in a manner regulated by the local stem cell niche (49). Although absorptive enterocytes are the most abundant cell subset, IECs form a heterogeneous network of cells with distinct functions. For example, secretory Paneth cells and goblet cells produce anti-microbial peptides and mucins, respectively, while M cells transcytose antigens across the intestinal epithelium to the Peyer's patches beneath. Enterocytes also produce a restricted repertoire of anti-microbial peptides throughout the GI tract, such as the C-type lectin regenerating islet-derived protein III-γ (REGIIIγ). The ability of IECs to form an effective barrier depends on their ability to act as frontline sensors of microbial stimuli through their expression of pattern recognition receptors (PRRs), including Toll-like receptor (TLR)5 and nucleotide-binding oligomerization domain (NOD)-like receptors (50–52). In turn, IEC-derived cytokines play essential roles in shaping the microbial structure of the GI tract, as well as the activation state of local immune cells during both homeostasis and inflammation (50, 53, 54). For example, interleukin (IL)-18 (50), IL-25 (55), thymic stromal lymphopoietin (56), and B cell-stimulating factors, including a proliferation-inducing ligand (APRIL) (57), are induced by PRR signaling in IECs and act to regulate local immunity. The importance of the epithelium in intestinal health is illustrated by genetic polymorphisms linking epithelial health and effector function with susceptibility to IBD and systemic inflammatory diseases, as shall be discussed later (58, 59).

**Figure 3:**
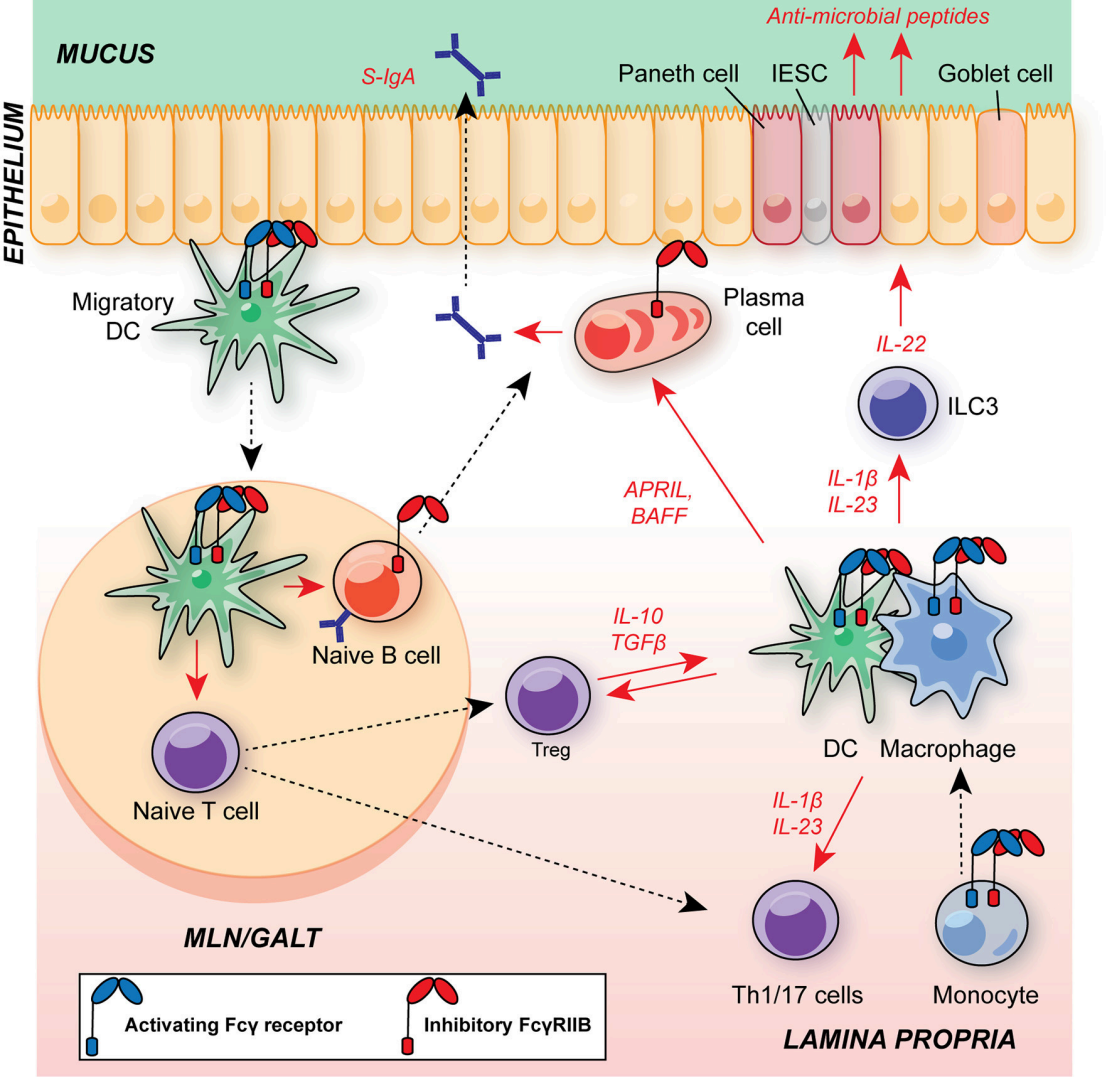
Schematic of major leukocyte populations in the intestine during homeostasis and their expression of FcγRs. Migratory DCs promote B cell IgA class-switching and T cell polarization in GALT and MLNs, including regulatory T cells (Treg) and Th17 cells. The balance of Tregs and Th17 cells is critical for maintaining intestinal homeostasis and suppressing excessive responses to the microbiota. CD11b^+^ cDC2s have an A:I ratio skewed toward FcγRIIB expression, as observed in DCs in other organs. FcγR-expressing DCs and monocyte-derived macrophages within the lamina propria support T cell and ILC3 activation via commensal-dependent production of cytokines, including IL-1β and IL-23. Tissue-resident macrophages are dependent on IL-10 for their suppressive function. APRIL and BAFF production by DCs and macrophages supports local plasma cell survival. Macrophage A:I ratio is skewed toward activating FcγRs and tuneable to the local milieu. Plasma cells and B cells express FcγRIIB, regulating survival and BCR activation threshold in these cells, respectively. Blue receptor, activating FcγR; red receptor, inhibitory FcγRIIB; S-IgA, secretory IgA; MLN, mesenteric lymph node; GALT, gut-associated lymphoid tissue; DC, dendritic cell; APRIL, a proliferation-inducing ligand; BAFF, B cell activating factor; IESC, intestinal epithelial stem cell; ILC3, group 3 innate lymphoid cell.

Beneath the epithelium, the intestinal mucosa and submucosa houses a range of innate and adaptive immune cells (Figure 3). Tissue-resident macrophages and dendritic cells (DCs) promote tolerance to intestinal commensal via anti-inflammatory cytokine production (including IL-10, and TGFβ, see section below). In addition, there are substantial populations of intestinal T cells, and the balance between regulatory T cells (Tregs) and T helper (Th)17 cells plays a fundamental role in determining whether there is mutualism with commensals or if their presence provokes inflammation. This is of particular interest, given the association of dysregulated IL-23/type 17 responses in the pathology of IBD and their role in defense against enteropathogens, such as *Citrobacter rodentium* (60–65).

IL-17A production by Th17 cells, γδ T cells and group 3 innate lymphoid cells (ILC3s) at epithelial barriers sites can play a central role in intestinal inflammation (66–69). IL-17 promotes neutrophil recruitment to tissues via the induction of CXCL1, CXCL2, and CXCL8, while IL-17A-induced granulocyte-macrophage colony-stimulating factor (GM-CSF) and matrix metalloprotease can sustain neutrophil activation and survival (70–72). IL-17 can also induce expression of anti-microbial peptides (73) and promotes the maintenance of the intestinal epithelial barrier (74–76), therefore IL-17 has both pro-inflammatory and homeostatic roles. The IL-17-inducing cytokine IL-23 can also stimulate interferon (IFN)γ secretion by inflamed colonic lamina propria cells (77), which in turn contributes to inflammation through the activation of tissue-resident macrophages, apoptosis of IECs, and augmentation of antigen processing (63). Studies utilizing IFNγ-deficient mice or IFNγ-depletion confirm its contribution to intestinal inflammation in murine models (78–80).

IL-10 is the prototypical anti-inflammatory cytokine. Most classically associated with Foxp3^+^ Tregs, IL-10 functions to suppress lymphocyte and myeloid immunity through a variety of mechanisms. Within the GI tract, the critical role of this cytokine is acutely illustrated by associations between *IL10R* single nucleotide polymorphisms (SNPs) and UC, as well as the widespread use of the *Helicobacter hepaticus* infection model: the presence or absence of IL-10R signaling dictates whether an otherwise tolerated bacterium induces colitis (81–83). Beyond Tregs and macrophages, regulatory B cells and epithelial cells also contribute to the intestinal IL-10 pool (84–86). Indeed, Rosser et al. demonstrated that microbiota-dependent IL-6 and IL-1β production was sufficient to induce IL-10-producing B cells within mesenteric lymph nodes (MLNs) that contain systemic inflammation (85), while adoptive transfer of these cells is sufficient to suppress intestinal inflammation (86).

## Antibodies in the Gastrointestinal Tract

### IgA in the GI Tract

While constituting only 10–15% of total serum Ig, IgA is the major antibody isotype at mucosal surfaces (87). Of the two major isoforms in humans (IgA1 and 2), IgA2 is associated with mucosal sites and has enhanced resistance to proteolysis. The intestinal epithelium has the capability of transporting of Ig into the lumen via expression of the polymeric immunoglobulin receptor (pIgR). Following pIgR binding and internalization at the basolateral IEC surface, dimeric IgA is released at the apical membrane by proteolytic cleavage of the secretory component of pIgR, forming secretory IgA. Once within the lumen, secretory IgA participates in the maintenance of mutualism through mechanisms collectively known as *immune exclusion*. This includes the ability to neutralize toxins and pathogens in the absence of complement fixation (88), the anchoring and agglutination of microbes within the mucus layer (89), as well as the recently described ability to “enchain” dividing bacterial daughter cells to limit microbial colonization and evolution, and promote elimination from the body (90). Natural or low-affinity IgA can also deliver antigens to M cells for transport into Peyer's patches for the induction of adaptive immune responses (91).

Gut-draining MLNs, gut-associated lymphoid tissues (GALT), including Peyer's patches and isolated lymphoid follicles, and the intestinal lamina propria itself have been proposed to be sites for IgA generation, with the mode of IgA generation differing across tissues (87, 92). Local IgA production within the lamina propria and isolated lymphoid follicles is thought to be largely T cell-independent, given the absence of abundant T cell zones, instead relying on B cell-intrinsic TLR signaling and secretory factors released by DCs and non-haematopoietic cells (57, 93). The MLNs, caecal patch, and Peyer's patches can support both T cell-dependent and -independent IgA induction, with the former linked to invasive species of commensals and pathobionts (94–97).

### IgG in the GI Tract

IgG1^+^ and IgG2^+^ plasma cells are present in the human gut. Both polyreactive and antigen-specific responses have been identified that target both foreign (commensals and enteropathogens) and self-antigens (10, 98). All antibodies show evidence of antigen-mediated selection and somatic hypermutation, with little difference in antigen reactivity between IgG isotypes, and no evidence of clonal relation between IgG and IgA responses within donors.

IgG2b and IgG3-producing B cell populations were also identified in the Peyer's patches and MLN of healthy adult mice, consistent with human observations. Murine IgG was elicited primarily by commensal-driven TLR signaling on B cells in a T cell-independent manner. Despite the relatively lower concentration of mucosal IgG compared with IgA, intestinal IgG responses exhibit broad microbial reactivity. Furthermore, most commensals bound by IgA antibodies in the murine gut also engage serum IgG2b and IgG3, while certain species of commensal microbes are uniquely opsonized by IgG3 across bacterial phyla (7).

The signals that lead to the local generation of IgG within the gastrointestinal tract have not been completely defined. However, *Escherichia coli* LPS has been shown to induce the B cell enzyme activation-induced cytidine deaminase (AID) and T cell-independent class-switch recombination to IgG2b and IgG3 in mice through dual B cell receptor (BCR)/TLR4 signaling (99, 100), and this may contribute to IgG generation in GALT. Indeed, a subset of IgG3- and IgG2b-expressing B cells within the Peyer's patches and MLN express the B1 cell marker CD43, a B cell subclass specializing in T cell-independent IgG production (7). T cell-dependent IgG responses have also been reported from B2 and follicular B cells in the GI tract, although the nature of the signals dictating T cell dependency remains to be determined. Higher affinity T cell-dependent responses may be reserved for invasive or epithelium-adherent species, in a manner analogous to IgA (90, 101) and consistent with the role of immune IgG in protection from enteropathogens (6, 102). Natural antibodies of the IgG3 subclass have also been described in mice and shown to interact with ficolins and mannose binding lectin that bind to bacteria, leading to FcγR-mediated phagocytosis by monocytes and inflammatory cytokine production (103).

### Fcγ Receptors and Their Expression in Immune Cells in the GI Tract

FcγRs are expressed by a number of immune cells within the GI tract, conferring the ability to respond to local IgG IC, and we will consider each cell type in turn:

#### Intestinal Macrophages

Macrophages play a central role in tissue homeostasis and inflammation, with increasingly appreciated non-immune roles in tissue function. Within the GI tract, macrophages are found throughout the lamina propria, the connective tissue that underlies the intestinal epithelium, as well as between the circular and longitudinal muscle layers (104–106). Here, they are continuously exposed to microbial stimuli and play a central role as key guardians of the homeostatic milieu through their potent phagocytic capacity and ability to produce an array of cytokines and chemokines.

Elegant studies by Bain et al. demonstrated that adult intestinal macrophages are largely derived from CCR2-dependent Ly6C^hi^ monocyte recruitment, a process dependent on the microbiota (107). Although intestinal macrophage numbers are unaltered in germ-free mice (108), commensal microbes influence macrophage activation state (109), for example via the production of short chain fatty acids (SCFAs) (110).

In homeostasis, intestinal macrophages are skewed toward anti-inflammatory functions, including the basal production of IL-10 (104, 111, 112). Indeed, macrophage-derived IL-10 can dampen IL-23-mediated colitis and promote regulatory T differentiation *in vitro* (111, 113).

During colitis, infiltrating monocytes are redirected toward a more pro-inflammatory macrophage phenotype (112, 114), with elevated production of IL-1β, IL-23, and tumor necrosis factor (TNF). These, in turn, promote Th1/Th17 and innate lymphoid cell (ILC) activation. Thus, inflammatory macrophages are the principle mediators of tissue inflammation in several murine models of intestinal pathology (60, 115–119).

Intestinal macrophages express a number of cell surface receptors, including TLRs, C-type lectin receptors, NOD-like receptors, and FcγRs (120). Indeed, FcγRI (CD64) is often used to discriminate between macrophages (FcγRI^+^) and dendritic cells (FcγRI^−^) in the GI tract (121). Studies in human monocyte-derived macrophage and murine bone marrow derived macrophages demonstrate expression of all canonical FcγRs, and expression is tuneable to the local environment: Th2 cytokines, such as IL-4, upregulate the expression of FcγRIIB and decrease the so-called activating/inhibitory ratio on macrophages (122–124). Generally, however, FcγR expression is skewed in favor of activating signaling (125, 126). A comprehensive analysis of FcγR expression in human gastrointestinal macrophages, across different anatomical regions of the gut, and in health and disease has yet to be performed.

FcγR cross-linking *in vitro* induces a potent macrophage inflammatory response, characterized by the production of IL-1β, IL-6, IL-10, IL-12, and TNF-α, as well as chemokines including CXCL8 (127). Interestingly, several studies have highlighted a link between TLR and FcγR co-stimulation and the induction of a Th17 polarizing macrophage phenotype. Stimulation of human M2 macrophages and DCs with IgG-opsonized bacteria induces IL-1β and IL-23 expression (128–130). Given the role of these same macrophage-derived cytokines in intestinal pathology, FcγR cross-linking by anti-commensal IgG signaling could contribute to Th17 cell activation and inflammation in the human gut. However, some murine studies suggest that IgG immune complexes can inhibit LPS-induced IL-1β and TNF production in BMDMs, a phenomenon at least partly dependent on prostaglandin E2 (PGE2) production (131, 132). Therefore, further work is needed to understand whether *in vitro-*derived macrophages accurately reflect tissue macrophage activation by IgG immune complexes *in vivo*, particularly in the complex environment of the gastrointestinal tract.

#### Intestinal Dendritic Cells

DCs sit at the interface of innate and adaptive immunity, their primary function being the transport of antigen from peripheral sites to draining lymph nodes in a CCR7-dependent manner for MHC-dependent presentation to T cells. Additionally, DC-derived cytokines play key roles in shaping innate and adaptive immune responses, including T cell polarization, B cell class-switch recombination, and innate lymphoid cell activation (Figure 3) (105, 133).

There are two main subsets of classical or conventional DCs (cDCs) derived from DC-specific precursors: BATF3-dependent cDC1s and IRF4-dependent cDC2s (134). In the murine intestine, several DC subsets have been characterized that variably express CD11b and CD103, with unique functions in health and disease (105). CD103^+^ CD11b^−^ cDC1s predominantly reside within GALT and are specialized in cross-presentation, while CD103^+^ CD11b^+^ cDC2s are found within the lamina propria and migrate to the MLN for presentation of exogenous antigens to CD4^+^ T cells and promote humoral immunity. CD103^+^ CD11b^+^ cDCs are essential for IL-6-dependent Th17 generation in the small intestine and colon (135) and necessary for the generation of IgG- and IgA-class-switched B cell responses to flagellin in MLNs (136). CD11b^+^ CD103^−^ DC subsets have also been described with the capacity to induce IL-17A and IFNγ production by T cells (137, 138). CD103^+^ DCs have also been identified as major sources of IL-23 in the context of *Citrobacter rodentium* infection, a widely used model of human attaching-effacing *Escherichia coli* infection (139–141). CD103^+^ DCs are also essential for the maintenance of tolerance via the induction of gut-homing CCR9^+^ FoxP3^+^ Tregs and IgA^+^ plasma cells, both within MLNs and GALT (88, 97, 133, 142).

DCs express FcγRI and FcγRIIA in humans, and FcγRIII in mice, although FcγR expression is skewed toward inhibitory FcγRIIB in immature DCs. Indeed, FcγRIIB blockade leads to spontaneous DC maturation and the induction of a cytokine programme characterized by TNF, IL-6, CXCL8, and IL-12p70 production (127, 143, 144), although as with macrophage studies, these data are generated on *in vitro* differentiated DCs. Analysis of Immgen consortium datasets on *in vivo* differentiated DCs identifies FcγR expression with cDC2s (126). This expression is tightly regulated, with maturation signals, such as LPS or IFNγ, down-regulating FcγRIIB to allow for IgG-induced cell activation via activating FcγR ligation. DCs efficiently process FcγR-internalized antigen and upregulate MHC and co-stimulatory molecules for robust antigen presentation to T cells (145–147). Furthermore, FcγR cross-linking on DCs induces CCR7 and matrix metalloprotease expression that facilitates their migration from inflamed peripheral sites to local draining lymph nodes (148). As well as its role in IgG recycling, FcRn has also been demonstrated to mediate the presentation of immune complexed antigens by DCs; FcRn engagement by immune complexes protects antigens from degradation following FcγR-mediated internalization, preserving them for presentation and cross-presentation to CD4 and CD8 T cells, respectively (149). Therefore, classical FcγRs and FcRn play a key role in DC antigen presentation to T cells through a variety of mechanisms.

As with macrophages, comprehensive information on FcγR expression in human gastrointestinal DCs across different anatomical regions of the gut is currently lacking.

#### Intestinal Neutrophils

The most abundant circulating leukocyte, neutrophils are first line inflammatory responders specializing in anti-microbial defense. This includes microbial internalization and killing, the release of proteases and reactive oxygen species, cytokine production, and the formation of neutrophil extracellular traps (NETs) (150). These functions are critical for defense following barrier breach, but their potent pro-inflammatory capability inevitably means that neutrophils may also contribute significantly to collateral tissue damage during inflammatory responses. Beyond microbicidal activity, neutrophils can shape adaptive immunity, for example via the production of APRIL and B cell activating factor (BAFF) in the marginal zone of the spleen (151), and may even be able to present antigen to T cells (152–154) identifying neutrophils as a more versatile immune subset than previously appreciated.

Neutrophils have long been associated with inflammation in patients with IBD and in defense against enteropathogens, rapidly recruited to the mucosa by resident and inflammatory mononuclear phagocytes (MNPs) (155, 156). However, their role in intestinal inflammation remains enigmatic, exhibiting both detrimental and protective functions in a context-dependent manner (157–159), including the production of barrier-protective IL-22 (160). The extravasation of neutrophils into the intestine is also observed during homeostasis, where their engulfment by resident MNPs suppresses *Il23a* induction (161).

Neutrophils constitutively express FcγRIIA and FcγRIIIB in humans, and FcγRIII and FcγRIV in mice, with low levels of FcγRIIB in both species (162–164). FcγR cross-linking has profound effects on neutrophil function, as expected of cells acutely poised to respond to stress. This includes NET formation (165), cytokine production, such as IBD-associated TNF and oncostatin M (166, 167), endothelial cell adhesion (168), phagocytosis (169), and reactive oxygen species production (170). Unsurprisingly, therefore, polymorphisms affecting FcγR function have consequences for the IgG-mediated activation of neutrophils. Most notably, three variants of FcγRIIIB exist (NA1, NA2, SH) that exhibit differential activity. NA1 is associated with enhanced uptake of IgG-opsonized erythrocytes (171), while SH enhances surface FcγRIIIB expression (172). Furthermore, copy number variation in *FCGR3B* correlates with FcγRIIIB surface levels, IC uptake, and soluble serum FcγRIIIB (164).

#### B Cell Response

As well as being the source of antibodies, B cells are also themselves regulated by IgG via expression of FcγRIIB, where it cross-links to the BCR to increase the cellular activation threshold and suppress antibody production (18). Furthermore, direct cross-linking of FcγRIIB on the surface of mature B cells and bone marrow-resident plasma cells can directly mediate apoptosis, thereby limiting the peripheral pool of antibody-producing cells (173, 174). As such, FcγRIIB has a critical role in maintaining humoral tolerance. Whether local IgG-IC engagement of FcγRIIB in GALT similarly regulates B cells in the gastrointestinal tract is currently undetermined.

#### Innate Lymphocytes

ILCs are a recently identified class of immune cells that are enriched at mucosal sites, such as the GI and respiratory tracts (175). ILCs subsets mirror those seen for T cells. Natural killer (NK) cells represent the innate cytotoxic equivalent of CD8^+^ T cells, whereas non-cytotoxic *helper* ILCs resemble CD4^+^ T helper cells. All helper ILCs, hereafter referred to simply as ILCs, express IL-2Rα and IL-7Rα, but unlike T cells and B cells, they do not express somatically-rearranged antigen-specific receptors (176). Helper ILC subsets are divided into ILC1s, ILC2s, and ILC3s that largely mirror the transcription factor-dependence and cytokine effector profile of Th1, Th2, and Th17 cells, respectively.

Given the importance of Th17 biology in the gut, it is unsurprising that the GI tract represents one of the major sites of ILC colonization. Mature NKp46^+^ ILC3s reside primarily in the intestinal mucosa and require RORγt for their development (177–179). Lymphoid tissue inducer (LTi) cells primarily reside within SLOs and represent a distinct ILC3 subset that develops partly independently of other helper ILCs (180). They contribute to lymphoid organogenesis and regulate local immunity through MHC-II expression and cytokine production (181–184). Through their production of IL-22, ILC3s play a critical role in the reinforcement of the intestinal epithelial barrier. Early studies demonstrated that *C. rodentium* infection induced innate intestinal IL-22 production that was dependent on IL-23 and the microbiota (117, 185). Subsequently, several studies have shown that IL-22R signaling mediates REG family AMP production, IEC fucosylation, and epithelial stem cell proliferation (64, 186–191). ILC3s are also an important source of GM-CSF, a key regulator of tissue immunity. In complementary studies, ILC3-derived GM-CSF was shown to orchestrate monocyte and granulocyte recruitment to the inflamed large intestinal lamina propria in anti-CD40 and *H. hepaticus* models of colitis (192–194).

FcγR expression has not been extensively investigated in ILCs. Their cytotoxic counterparts, NK cells, express activating FcγRIIC and FcγRIIIA in humans, and FcγRIII in mice, but not FcγRIIB (195, 196). FcγR signaling on NK cells stimulates the targeted release of cytotoxic molecules to kill opsonized cells, a process known as antibody-dependent cell-mediated cytotoxicity (ADCC), as well as IFNγ and TNF-α release (197, 198). Several lines of evidence suggest that FcγR signaling may impact ILC function, either directly or indirectly. Firstly, FcγR signaling on MNPs may drive ILC3 activation through the production of type 17-inducing cytokines, such as IL-1β and TL1A (129, 199, 200). Secondly, FcγRIII expression has been identified in murine ILC3s in global transcriptomic studies (201, 202). Therefore, FcγR expression may be a common feature of cytotoxic and helper ILC subsets.

## Intestinal IgG in Health and Pathogen Defense

### Neonatal Immunity

The acquisition of maternally-derived secretory antibodies *in utero* and through breast milk provides neonates with an important source of immunity prior to the development of the host immune system (203). Mucosal pathogens are a major cause of death in children below the age of 5, with epidemiological data indicating that breastfeeding confers a 20-fold reduction in infant mortality from diarrhea. In this setting, maternally-derived IgG plays a key role in the maintenance of mutualism between the neonate and microbiome, tolerance toward innocuous dietary and environmental antigens, and systemic protection from pathogenic challenge (5–8, 204, 205).

**Figure 4:**
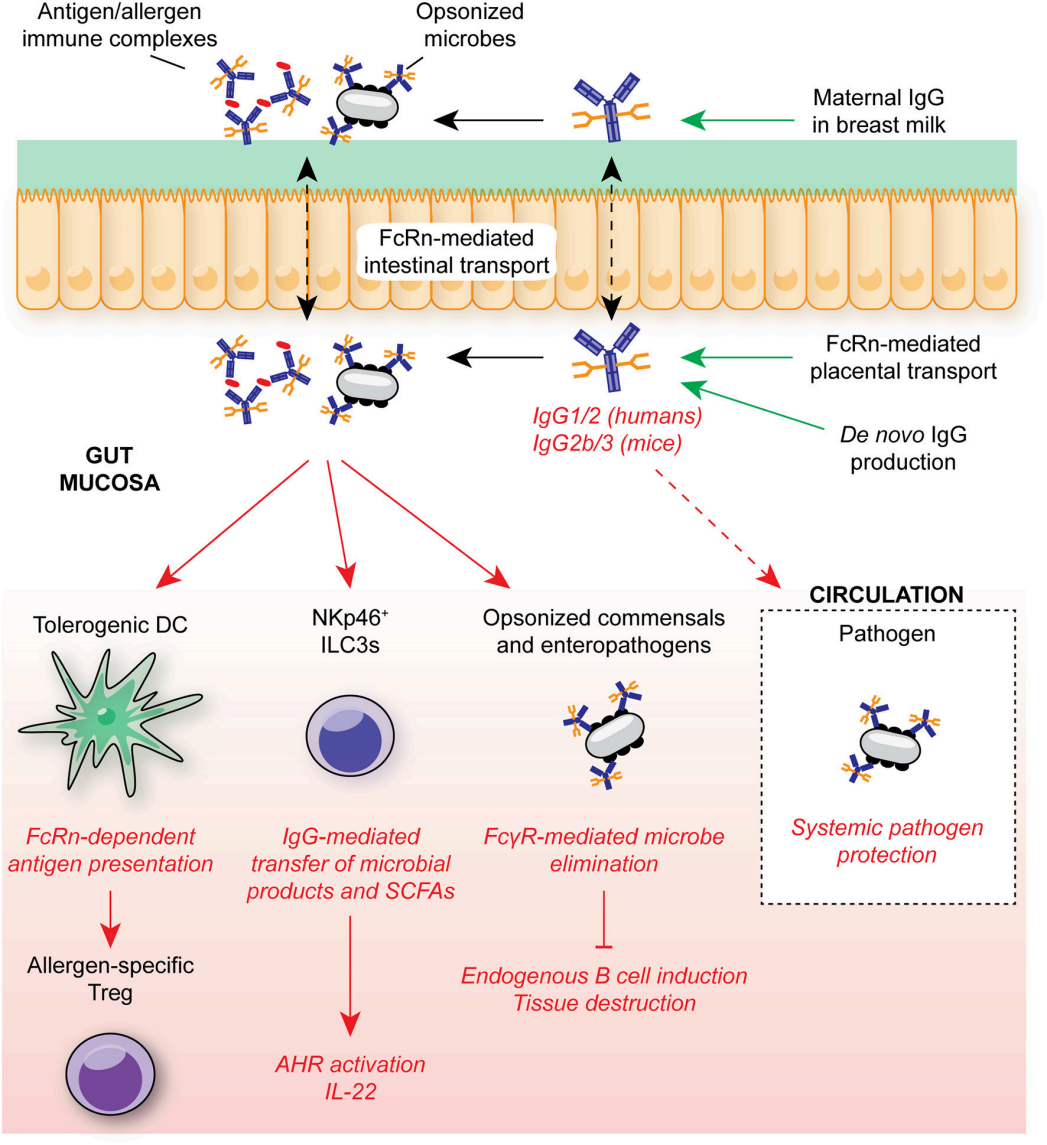
IgG in intestinal homeostasis. FcRn-mediated placental and epithelial transport contributes to the neonatal anti-microbial IgG repertoire in early life, mediating protection against opportunistic mucosal invasion. Maternally-derived IgG contributes to protection from allergic responses through FcRn-mediated antigen presentation by DCs to T cells for regulatory T cell induction. IgG-mediated transfer of microbial molecules, such as SCFAs, also supports appropriate immune cell development. In adult humans and mice, anti-microbial IgG is generated throughout life in GALT, contributing to systemic protection from infection through engagement of FcγRs on myeloid cells. In humans, FcRn is continuously expressed within the intestinal epithelium, allowing for bidirectional trafficking of IgG and immune complexes between the intestinal lumen and lamina propria for antigen delivery to local FcγR-expressing myeloid cells. SCFA, short-chain fatty acids; ILC3, group 3 innate lymphoid cell; FcRn, neonatal Fc receptor; AHR, aryl hydrocarbon receptor.

Koch et al. demonstrated that in the absence of intestinal FcRn-mediated IgG uptake, neonates exhibited enhanced bacterial translocation to the MLN and compensatory mucosal inflammatory responses, most prominently exacerbated T follicular helper responses and germinal center B cell activation (Figure 4) (7). This response was subsequently superseded by *de novo* anti-microbial IgG generation after weaning. Similarly, maternally-derived intestinal IgG was demonstrated to mediate protection against *Heligmosomoides polygyrus*, both through luminal delivery via milk and FcRn-mediated epithelial transport from the circulation (206). In a model of commensal colonization, Macpherson and colleagues further demonstrated the requirement for maternal IgG antibodies in the development of tissue-resident immune cells in neonates. Neonatal expansion of small intestinal NKp46^+^ ILC3s and IL-22-dependent genes, such as REGIIIγ, was IgG-dependent, driven by the retention and subsequent transfer of microbe-derived metabolites to offspring as immune complexes (5) (Figure 4). Strikingly, this included ligands for AHR, a key transcription factor mediating ILC3 development (178, 207). Consequently, offspring to colonized dams were better able to control intestinal microbial challenge and suppress systemic dissemination of bacteria.

Beyond microbial recognition, intestinal IgG plays a key role in tolerance to innocuous antigens. In a recent elegant study, Ohsaki et al. demonstrated that epicutaneous sensitization of pregnant mothers with model allergens resulted in suppressed allergic responses in offspring subsequently challenged with the same antigen (8). Lower allergen-specific IgE titres, impaired mucosal and systemic Th2 activation, and reduced anaphylaxis was attributed to intestinal FcRn-mediated allergen:IgG immune complex uptake and presentation within MLNs by tolerogenic DCs which, in turn, induced protective mucosal Tregs. Indeed, given the roles for FcγR signaling in DC activation, this suggests IgG-mediated trafficking and presentation may be a common mechanism of immune complex-mediated immune responses in DCs across tissues (148). Whether similar mechanisms exist for the induction of microbe-induced Tregs remains to be established. Furthermore, how DC presentation of IgG-opsonized antigens may be altered during mucosal inflammation, and whether this induces pro-inflammatory T cell responses that promote microbial elimination has yet to be explored.

### Intestinal and Systemic Infection

In healthy adult mice, microbe-specific IgG is largely excluded from the intestinal lumen, suggesting that its major site of action is in the intestinal wall, where it combats invasive microbial species, and in protection from systemic challenges. For example, IgG responses induced by gram negative bacterial antigens have been demonstrated to confer FcγR-mediated protection against systemic *E. coli* and *Salmonella* challenge (205). Indeed, murine IgG2b is known to effectively engage FcγRs and complement receptors, suggesting a role in the activation of mucosal phagocytes (208). A striking aspect of this response is its inherent flexibility, tuneable to fluctuations in microbial loads and to genetically-determined variation in the strength of the mucosal innate immune system. For example, defective TLR signaling or oxidative burst production augments gut T cell-dependent microbial IgG titres that act to preserve mutualism *in vivo* (204). Therefore, IgG-mediated anti-commensal responses represent an essential and plastic mechanism required to maintain host-microbe mutualism and mediate protection from systemic pathogen spread.

The context of antigen delivery to GALT is a critical determinant of subsequent humoral immune responses. Antigen presentation in inflamed or infected mucosae, or from high antigenic loads, induces potent antibody responses due to alterations in adjuvant-derived signals, leading to high-avidity T cell-dependent antibodies that contribute to specific elimination of the inductive bacterial species (90, 209). As well as being induced continuously in GALT largely independently of T cells, *de novo* T cell-dependent IgG has been identified in models of intestinal infection, where it has been shown to make essential contributions to sterilizing immunity.

In murine models, B cells are required for clearance of *C. rodentium*, although this was independent of secretory IgA and IgM (102). Although initial disease activity and bacterial containment was equivalent between B cell-sufficient and deficient mice at 2 weeks post-infection, B cell-deficient mice exhibited enhanced mucosal inflammation and severe crypt hyperplasia with ulceration at 6 weeks, consistent with defective infection control. Disease susceptibility was rescued only by passive transfer of IgG-replete immune serum (210). Anti-*C. rodentium* IgG titres are diminished in CD4^+^ T cell-deplete mice, which are highly susceptible to *C. rodentium*, suggesting a critical role for T cell-dependent antibody responses (211). Adequate IgG responses to *C. rodentium* are also dependent on NOD2-mediated bacterial sensing within the intestinal epithelium. Impaired local adaptive immune activation is observed in *Nod2-*deficient mice, attributed to reduced CCL2-mediated monocyte recruitment (212). Monocyte-derived IL-12 was required for induction of Th1 cells (213), with IFNγ being an effective driver of IgG class-switching (214, 215), suggesting a possible mechanism for this effect. As well as complement-fixing activity (216), the contribution of FcγRs to IgG-mediated protection in this model was investigated using Fc receptor common gamma chain (FcRγ)-deficient mice, which lack productive signaling from activating FcγRs (217). FcRγ-deficient mice phenocopy B cell-deficient mice, succumbing more rapidly to infection, with increased bacterial burden and mucosal inflammation. In the absence of FcγR signaling, MNP-mediated *C. rodentium* phagocytosis, cellular maturation, and inflammatory cytokine production was impaired, as was antigen presentation to T cells. In an elegant study, Kamada et al. demonstrated the specific targeting of virulence factors on invasive strains of *C. rodentium* by mucosal IgG, consistent with their ability to adhere to the intestinal epithelium and elicit inflammation (6). In contrast, non-virulent strains and commensals residing predominantly within the lumen remained untargeted. Both MNPs and neutrophils are required for FcγR-mediated protection, with neutrophils shown to directly access opsonized bacteria within the intestinal lumen (6). Furthermore, more recent work from Caballero-Flores et al. has demonstrated that this IgG-mediated protection against *C. rodentium* can be vertically transmitted to offspring via breast milk (218).

In humans, FcRn expression is not limited to a brief developmental window, allowing the entry and retrieval of IgG from the intestinal lumen throughout health and disease. The importance of this was demonstrated in transgenic murine experiments with forced expression of human FcRn in murine IECs (26). This bidirectional transport allowed the secretion of IgG into the lumen, the subsequent uptake of opsonized bacteria, and the induction of local antigen-specific CD4^+^ T cell responses required for clearance. Therefore, cross-talk between CD4^+^ T cells and IgG-expressing B cells appears to be required for effective intestinal pathogen clearance. FcRn-mediated protection has also been demonstrated in a mouse model of *Helicobacter pylori* infection, with reduced IgG levels in gastric juice of challenged FcRn-deficient animals, with increased bacterial penetrance and activated lymphoid follicles compared to controls (219).

Beyond models of murine bacterial challenge, several studies in non-human primates have also identified roles for IgG in anti-viral mucosal immunity. Passive administration of anti-HIV neutralizing IgG can prevent mucosal viral transmission in rhesus macaques following oral administration (220, 221). The Fc domain of anti-HIV broadly neutralizing antibodies was required for anti-viral activity *in vivo* (222) and could be further enhanced through Fc domain engineering to augment Fc-mediated activating FcγR engagement (222). Fc domain modification to increase FcRn binding also increased the serum half-life of the antibody and enhanced mucosal tissue localization (223). In a recent study, it was demonstrated that the site of immunization influences the dominant protective mechanisms of elicited Igs (224). Intramuscular vaccination induced an IgG-dominated response, with protection correlating most strongly with FcγR-mediated viral phagocytosis by monocytes. In contrast, mucosal vaccination via aerosol elicited an IgA-skewed response that correlated with neutrophil-mediated phagocytosis. Protection via mucosal vaccination also correlated significantly with FcγRIIA binding, suggesting that mucosal vaccine-specific IgA and IgG may cooperate to drive neutrophil-mediated viral clearance *in vivo*. Despite these differences in mechanism, both routes were equally effective in suppressing viral infection. Similarly, passive transfer of serum rotavirus-specific IgG has been shown to suppress oral rotavirus infection in naïve pigtailed macaques (225).

## IgG, FcγRs, and Intestinal Inflammation

### Inflammatory Bowel Disease

Inflammatory bowel disease is a chronic relapsing inflammatory disorder of the GI tract that causes considerable morbidity and is associated with an increased risk of colonic cancer (156, 226, 227). There are two main subtypes, Crohn's disease (CD), and UC, that differ in their clinical and pathological presentations. CD may affect any part of the GI tract and is associated with transmural inflammation affecting the entire mucosa and the formation of granulomas. In contrast, in UC, lesions are localized to the large bowel, resulting in continuous superficial mucosal inflammation and ulceration of the intestinal wall, with micro-abscess formation and neutrophil infiltration within the lamina propria common.

GWA studies (GWAS) have contributed significantly toward the understanding of the pathogenesis of IBD, with the immune system and its interaction with the microbiome lying at the core of disease susceptibility. Disease is driven by a genetic predisposition to aberrant mucosal immune responses toward the microbiota in the context of poorly-defined environmental factors, with unique and shared genetic traits between CD and UC (156). Furthermore, many disease-associated mechanistic pathways in IBD are shared with other extra-intestinal diseases, including ankylosing spondylitis and psoriasis (14, 228). A pathway implicated by genetic studies in both UC and CD is the IL-23/IL-17 cytokine axis (61). A SNP associated with an amino acid substitution in the cytoplasmic domain of IL-23R conferred significant protection against IBD. Several murine studies also support a pathogenic role for IL-23 in the intestine, ascribed to its ability to drive Th17-mediated inflammation (229–231). In human studies, antibodies against the common p40 subunit of IL-12 and IL-23 led to increased rates of clinical response in CD compared with placebo.

More generally, innate cytokine production is dysregulated in IBD, including TNF and IL-1β (63). This is notably reflected in the efficacy of anti-TNF IgG monoclonal antibodies in the treatment of human disease (156, 232). Furthermore, clinical trials are underway for the use of Anakinra (IL-1Ra), an IL-1β antagonist, in the treatment of severe treatment-resistant UC (IASO trial) (233, 234). In addition to its role in mediating neutrophil inflammatory responses (235), IL-1β has important roles in type 17 immunity in concert with IL-23, particularly in the expansion and maintenance of Th17 cells within inflamed tissues (236). Strikingly, Ghoresch et al. demonstrated that combined stimulation of naïve T cells with IL-23, IL-1β, and IL-6, in the absence of TGFβ, was sufficient to induce a pathogenic subset of Th17 cells (237), while dual IL-23 and IL-1β stimulation has been shown to bypass the requirement for CD28-mediated co-stimulation for the induction of human Th17 cells (238). Despite the advances in our understanding of how these cytokines drive T cell-mediated pathology in the gut, therapeutic targeting of the downstream effector cytokines has proved unsuccessful, or even detrimental, in the treatment of IBD, as observed with monoclonal antibodies targeting IFNγ or IL-17A (63).

**Figure 5:**
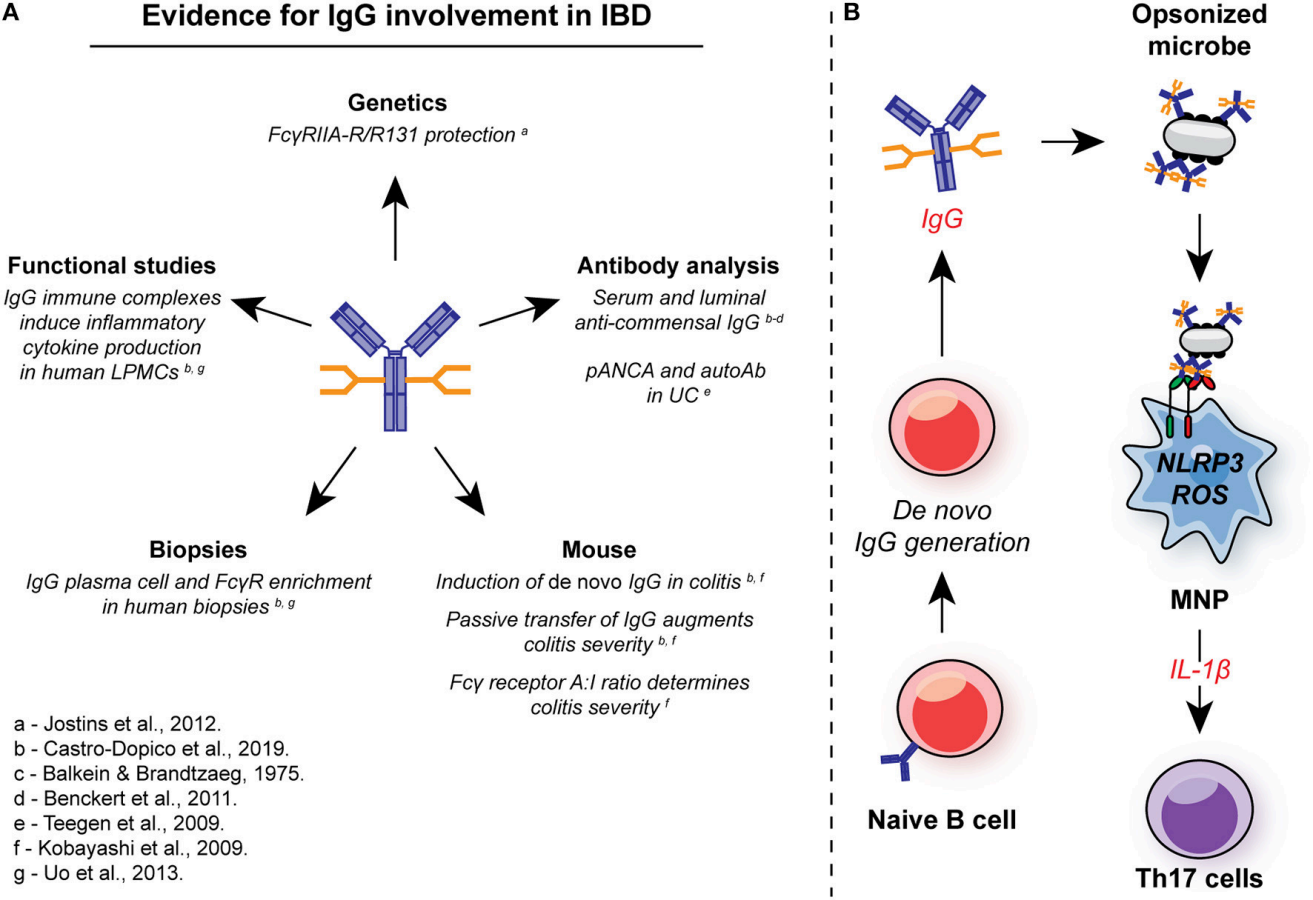
Involvement of IgG in intestinal inflammation. **(A)** There are multiple lines of evidence for the involvement of IgG and FcγR signaling in the pathogenesis in IBD. In genetic studies, the low-affinity FcγRIIA-R131 variant is associated with protection from UC in several independent cohorts. Systemic and local induction of *de novo* anti-commensal IgG and auto-antibodies, such as pANCA, is observed in human IBD, while IgG^+^ plasma cells and FcγR-expressing cells are enriched in mucosal biopsies from IBD patients with active disease. In murine studies, *de novo* IgG production is observed, and passive transfer of anti-flagellin IgG to naïve animals exacerbates DSS-induced colitis. FcγR signaling strength determines the magnitude of intestinal inflammation in this model. In humans, IgG immune complex stimulation of intestinal LPMCs drives inflammatory cytokine production, including IL-1β. **(B)** IgG and FcγR in intestinal inflammation: Inflammation is characterized by an increase in the generation of local anti-commensal IgG. These immune complexes cross-link FcγR on colonic MNP, leading to NLRP3 and ROS-dependent IL-1β production. IL-1β, in turn drives Th17 immunity propagating inflammation. ROS, reactive oxygen species; MNP, mononuclear phagocyte; LPMCs, lamina propria mononuclear cells.

*De novo* IgG generation has long been associated with chronic intestinal inflammation in IBD patients (9, 10, 98, 239) (Figure 5). IBD-associated IgG appears to be directed against components of the commensal microbiome, particularly flagellin (11, 239). A significant proportion of UC patients also develop of auto-antibodies. Perinuclear anti-neutrophil cytoplasmic antibodies (pANCA) are observed in two thirds of UC patients (240–242), while antibodies against colonic goblet cells have been observed in some subjects (243). CXCR4^+^ IgG plasmablasts have been described in the inflamed colonic mucosa of patients with IBD (200), suggesting local IgG production. However, despite these reports, a systematic characterization of the IBD-associated IgG repertoire and the identification of the cellular mechanisms by which IgG might contribute to IBD pathogenesis has been lacking.

Genetic studies in IBD support the thesis that IgG play a role in UC pathogenesis (Figure 5). A variant of FcγRIIA was initially associated with protection from UC in a Japanese case control study (13), and this was confirmed in a metanalysis of GWAS that included data from more than 70,000 patients. The FcγRIIA variant encodes a histidine or arginine at position 131 (H/R131) within the second Ig-like domain, resulting in variable ligand affinity (244, 245), with reduced binding affinity for IgG1 and IgG2 in the R131 variant receptor compared to the H131 receptor (31, 246), the major IgG isotypes detected in the human gut (10). In UC, homozygous R/R131 individuals are protected from disease (odds ratio = 0.63; *P* = 1.56 × 10^−12^) (13), suggesting that IgG plays a pathogenic role within the gut rather than being merely a bystander of inflammation. Of note, the FcγRIIA H/R131 SNP has been associated with susceptibility to a number of other autoimmune and inflammatory conditions. For example, the high-affinity H131 polymorphism is associated with Kawasaki disease and systemic vasculitis (247).

To further investigate the role of IgG in UC, we first assessed the extent to the fecal microbes were opsonized by IgA or IgG. In contrast to household controls, where <10% of luminal bacteria were bound by IgG, up to 80% of commensal microbes were IgG-opsonized in UC patients (248). In support of local production of this anti-commensal IgG, we found a marked increase in both heavy chain *Ig* gene transcripts within UC mucosal biopsies, as well as enrichment of several FcγR gene transcripts and associated signaling pathways. To investigate the pathological significant of these observations, we used dextran sodium sulfate (DSS)-induced colitis to interrogate the impact of IgG and FcγR cross-linking of intestinal inflammation. Kobayashi et al. previously demonstrated that exposure to DSS leads to *de novo* production of anti-flagellin IgG, a dominant IgG-targeted antigen in human IBD (249). We confirmed this and demonstrated a more widespread IgG response against multiple bacterial species, including both commensals and pathobionts. This was driven in part by the *de novo* emergence of IgG-producing B cells and plasma cells within the colonic mucosa and GALT, in line with previous observations (9, 98, 200). Immune cell profiling identified CX3CR1^+^ monocytes and macrophages as the major FcγR-expressing colonic cell types, including expression of the risk receptor FcγRIIA in humans. Furthermore, these cell subsets represent the dominant source of IL-1β production, one of the dominant inflammatory cytokines in human and murine colitis. Passive transfer of anti-flagellin IgG was sufficient to augment pro-IL-1β expression by colonic MNPs in mice exposed to DSS and enhance disease severity in a manner analogous to experiments by Kobayashi et al., whereby naïve mice exposed to anti-flagellin IgG exhibited enhanced weight loss and pathology scores. To assess the contribution of MNP FcγR signaling to this disease augmentation, we made use of transgenic mice with different FcγR A:I ratio through manipulation of the inhibitory receptor, FcγRIIB. *Fcgr2b*^−/−^ mice exhibited elevated MNP pro-IL-1β expression both *in vitro* and *in vivo*, leading to exacerbated colonic type 17 T cell responses, and impaired recovery from DSS exposure, effects mitigated by IL-1β blockade. In contrast, macrophage-intrinsic FcγRIIB over-expression imparted protection to DSS-induced colitis over non-transgenic littermate controls, demonstrating that MNP-intrinsic FcγR signal strength determines the magnitude of intestinal inflammation.

Our demonstration of the cellular and molecular mechanisms by which IgG-commensal immune complexes might drive inflammation in UC raise several additional questions. Genetic susceptibility studies in IBD to date have focused on *FCGR2A*, but variants in other FcγRs, particularly *FCGR2B*, may be of relevance to disease pathogenesis. For example, FcγRIIB-T232 (rs1050501) results in an isoleucine-to-threonine substitution in the receptor transmembrane domain of the receptor leading to exclusion of the receptor from sphingolipid rafts (250–252). FcγRIIB-T232 is associated with susceptibility to SLE (253, 254), and at a cellular level, with exacerbated pro-inflammatory responses to IgG immune complexes in macrophages and DCs (255). Conversely, the FcγRIIB-T232 genotype is associated with enhanced protection against some infections, including malaria (254). The activating FcγRIIIA-V158 variant, encoding a valine rather than a phenylalanine at position 158, exhibits enhanced affinity for all IgG subclasses (31) and is associated with susceptibility to rheumatoid arthritis (256, 257) and immune-mediated thrombocytopenic purpura. These SNPs in inhibitory FcγRIIB and activating FcγRIIIA have profound effects on IgG-mediated inflammation, and certainly have the potential to influence susceptibility to, or progression of, intestinal inflammatory disease. A comprehensive genetic profiling of *FCGR* polymorphisms in IBD will be required to address this question, but has previously been technically challenging due to the sequence similarity between the FcγR genes (that have arisen by gene duplication), and to the copy number variation at this locus (258).

A further interesting question raised by our study is whether abnormalities in the IgG glycome may be present in patients with IBD, and the extent to which these may influence the pathogenicity of anti-commensal IgG. Aberrations in IgG glycome have been described in several autoimmune disorders, and in individuals with impaired responses to infectious agents, such as *Mycobacterium tuberculosis*. The development of widescale IgG profiling via so-called “systems serology” by Alter and colleagues has further implicated distinct IgG glycosylation patterns with IgG functionality and immune reactivity in various disease settings (259–261). Increased agalactosylated IgG has been observed in patients with RA and SLE, a state which favors IgG binding to activating FcγRs, as well as a reduction in IgG sialylation (41, 262, 263). In IBD, abnormal patterns of IgG glycosylation have also been described, with increased agalactosylated IgG in both UC and CD (264), and decreased IgG sialylation detectable in CD (42, 265). Sialylated IgG is associated with increased binding to non-classical Fc receptors, such as DC-SIGN (45), the induction of FcγRIIB on effector cells (122), reduced complement-dependent cytotoxicity (266), and is an essential component of IVIg. Furthermore, agalacotsylated IgG exhibits reduced binding to FcγRIIB (38). Taken together, these data suggest that IgG profiles in IBD are skewed toward a pro-inflammatory phenotype, as observed in other antibody-mediated autoimmune diseases (19). Strikingly, five genes known to regulate IgG glycosylation show robust association with IBD (*IKZF1, LAMB1, MGAT3, IL6ST*, and *BACH2*) (42), including genes encoding galactosyltransferases (41). Given the association between pathological IL-23R signaling and IBD, and the observation that IL-23-derived Th17 immunity promotes IgG class-switching and inflammatory glycosyl patterns (267), these pathways may reinforce one another for the augmentation of pathology in the GI tract.

While the majority of studies focusing on FcγR signaling in GI immunity have focused on MNP and DC biology (200, 217, 248, 268), relatively little is known about how FcγR signaling in neutrophils may contribute to intestinal pathology. Neutrophils are massively expanded in IBD (155) and express FcγRIIA and FcγRIIIB, as well as lower levels of FcγRIIB, making them candidates to directly promote IgG-mediated inflammation. Furthermore, the emergence of pANCA IgG in two-thirds of UC patients directly implicates neutrophils in the ongoing intestinal humoral response (240–242). Indeed, increased *FCGR3B* gene copy number is associated with susceptibility to UC, directly implicating the FcγR-neutrophil axis in IBD (269). The mechanisms by which this axis leads to disease susceptibility, however, remain unexplored.

### IgG in Inflammation-Associated Intestinal Cancer

IBD is associated with a significant risk of developing colorectal cancer. Endogenous and therapeutic monoclonal antibody responses can contribute to tumor rejection *in vivo* through a variety of mechanisms, including DC-mediated T cell activation and NK cell-mediated ADCC (270, 271). Indeed, in the gut, anti-tumor immunity is impaired in the absence of functional IgG responses (268). Consistent with its ability to induce cross-presentation, FcRn-deficient DCs from the MLN of colitogenic mice were impaired in their ability to induce IFNγ production by OT-I cells *in vitro* (149), while MLN CD8 T cells from FcRn-deficient mice were equally impaired in their ability to produce granzyme B and IFNγ following *ex vivo* re-stimulation. In the context of tumorigenesis, DC-specific FcRn expression protected against the development of colorectal cancer and lung metastases in the *Apc*^*min*^^/+^ and DSS/azoxymethane (AOM) models via the homeostatic mucosal activation of endogenous tumor-reactive CD8 T cells (268). This protection was dually dependent on cross-presentation and IgG-IC-driven IL-12 production by DCs. In summary, FcγR-driven immune responses have the potential to contribute to both pathological and protective inflammation in IBD and cancer.

## FcγR Pathways in the Treatment of Intestinal Disease

### Targeting FcγR Signaling in IBD

Our demonstration of the mechanism by which anti-commensal IgG might drive intestinal inflammation in UC also has therapeutic implications for IBD, particularly given the finding that high levels of colonic IgG and activating FcγR receptor transcripts are associated with resistance to TNF blockade (248). Strategies aimed at reducing the production of pathogenic IgG or blocking its effector function via FcγRs, including B cell depletion, plasmapheresis, and IVIg administration, are commonly used in several inflammatory disorders but their application to IBD has not been studied extensively. No randomized control trials exist for IVIg but a meta-analysis identified a handful of case reports which indicated that IVIg can induce a rapid improvement in steroid resistant CD (272, 273), and there are reports of utility in UC (274). A single randomized controlled trial exists for the anti-CD20 antibody rituximab in UC, where the number of patients included precludes any robust conclusions (275). In UC, of 16 patients who failed to respond to standard therapies, half demonstrated a response at 4 weeks compared to 2 of 8 placebo-treated patients, although this was not maintained to 12 weeks in a further half of patients. However, this study was substantially underpowered, given that studies examining the efficacy of anti-TNF therapies contain hundreds of patients (234). The efficacy of rituximab in depleting mucosal B cells in this study is unclear, given the use of CD20 expression itself to determine depletion, which may be masked by rituximab. Rituximab leads to pan-B cell depletion, which can exacerbate allo- and auto-immunity due to removal of regulatory B cells (276–278) and may be a sub-optimal therapeutic strategy, although rituximab does not appear to exacerbate UC in this instance. Finally, the experimental design of this study does not investigate the effect of repeated B cell depletion, which is associated with long-term treatment response in RA (279). Therefore, the question of whether B cell manipulation may be of benefit in UC has not been adequately addressed.

FcRn inhibitors are currently under development for use in autoimmune diseases, and effectively reduce serum IgG (280–283). Given the prominent role for FcRn in the GI tract, this may also influence IgG epithelial transport and immune complex-mediated local T cell activation within the mucosa of IBD patients.

As well as targeting IgG generation, therapeutic manipulation of FcγR signaling may also prove effective in IBD. Although small molecule SYK inhibitors were shown to be beneficial in RA patients (284), off-target side-effects are common given the widespread expression and function of SYK (285). Modulation of FcγRIIB activity is central to many newer therapies. Enforced co-localization of FcγRIIB with CD19 on B cells using an engineered anti-CD19 monoclonal antibody successfully suppressed humoral immunity in peripheral blood mononuclear cell (PBMC)-engrafted SCID mice (286). Furthermore, small preliminary studies have demonstrated efficacy of soluble human FcγRIIB in the treatment of ITP and SLE (285). However, a deeper understanding of the mechanisms of IgG-mediated inflammation in the GI tract are required for the development of sophisticated therapeutic strategies.

### FcγR Influence on Therapeutic Intervention

Beyond targeting IgG-mediated inflammation therapeutically, it is known that FcγR polymorphisms can influence the efficacy of monoclonal antibody therapies in IBD. CD patients homozygous for the high affinity FcγRIIIA-V158 variant demonstrated improved biological responses (as determined by reduced CRP), and a trend toward improved clinical responses, to infliximab compared to FcγRIIIA-F158-bearing individuals (287, 288). NK cells and PBMCs from FcγRIIIA-V158 homozygotes exhibited increased antibody binding and ADCC in response to infliximab. Furthermore, FcγR-dependent effector function has been implicated in mediating protective functions of infliximab at least partly via its ability to form anti-inflammatory immune complexes with trimeric TNF (289, 290). Genetic variation in FcγRIIA can also influence responses to therapeutic monoclonal antibodies, for example the efficacy of rituximab therapy in B-cell lymphomas (291).

FcγR function also has a key role in the effector function of therapeutic monoclonal antibodies in tumor immunotherapy, including checkpoint blockade (292, 293). The specific contributions of FcγR-mediated effector functions have been uncovered through the generation of Fc-optimized antibodies. FcγR-optimized anti-CD25 IgG shows improved ability to deplete intra-tumoral Tregs by bypassing the upregulation of FcγRIIB by tumor-associated macrophages and cDCs (292). Similarly, anti-CTLA4 IgG requires an Fc domain for activity (294), with hIgG2 antibodies driving FcγRIIA-mediated intra-tumoral Treg depletion in humanized mice (293). Strikingly, FcγRIIIA co-engagement on APCs by anti-CTLA4 IgG also augmented APC-T cell interaction and promoted pro-tumoricidal effector T cell responses (295).

These considerations extend to colorectal cancer, where patients treated with cetuximab, a monoclonal IgG1 antibody directed against EGFR, displayed improved survival in the presence of the high-affinity FcγRIIA-H131 variant (296). However, despite these advancements, a significant proportion of patients remain unresponsive to treatment (293) and further studies are required to determine the impact of these FcγR-mediated effector functions in immunotherapy.

## Conclusion

Despite the identification of IgG positive cells in colonic biopsies more than 40 years ago by Baklien and Brandtzaeg (9) and the subsequent confirmation of the anti-microbial specificity of this mucosal IgG (239), research into the role of IgG antibodies in an otherwise IgA-dominated organ system has been relatively limited. However, the identification of FCGR2A^*^A519G (rs1801274) as the most-significant non-HLA genetic variant associated with UC in a Japanese GWAS (13), confirmed in candidate gene studies (odds ratio 0.70–0.84) (297, 298) and in a subsequent meta-analysis of IBD GWAS (14), has brought the potential role of mucosal IgG in inflammation into focus.

We and others have shown that microbial IgG forms a core component of the intestinal inflammatory response, both in models of IBD, such as DSS-induced colitis, as well as mucosal infection, potentially identifying novel therapeutic strategies for IBD. However, the role of IgG and FcγRs extend beyond local inflammatory responses and play essential roles in mucosal immune cell education, commensal regulation, oral tolerance, systemic immune protection, and cancer. Future studies will be required to elucidate the signals that determine the generation of these IgG responses in different settings and the mechanisms by which they contribute to local immunity.

## Author Contributions

All authors listed have made a substantial, direct and intellectual contribution to the work, and approved it for publication.

### Conflict of Interest Statement

The authors declare that the research was conducted in the absence of any commercial or financial relationships that could be construed as a potential conflict of interest.

## Abbreviations

ADCC: Antibody-dependent cell-mediated cytotoxicity
AHR: Aryl hydrocarbon receptor
AID: Activation-induced cytidine deaminase
APRIL: A proliferation-inducing ligand
BAFF: B cell activating factor
BCR: B cell receptor
CD: Crohn's disease
cDC: Classical/conventional DC
CRP: C reactive protein
DC: Dendritic cell
DSS: Dextran sodium sulfate
FcγR: Fcγ receptor
FcRn: Neonatal Fc receptor
GALT: Gut-associated lymphoid tissue
GI: Gastrointestinal tract
GlcNAc: *N-*acetylglucosamine
GM-CSF: Granulocyte-macrophage colony-stimulating factor
GWAS: Genome wide association study
IBD: Inflammatory bowel disease
IC: Immune complex
IEC: Intestinal epithelial cell
IFN: Interferon
Ig: Immunoglobulin
IL: Interleukin
ILC: Innate lymphoid cell
ITAM: Immunoreceptor tyrosine-based activating motif
ITIM: Immunoreceptor tyrosine-based inhibitory motif
IVIg: Intravenous immunoglobulin
MHC: Major histocompatibility complex
MLN: Mesenteric lymph node
MNP: Mononuclear phagocyte
NOD: Nucleotide-binding oligomerization domain
NET: Neutrophil extracellular trap
NK: Natural killer
pANCA: Perinuclear anti-neutrophil cytoplasmic antibodies
PBMC: Peripheral blood mononuclear cell
PGE2: Prostaglandin E2
PI3K: Phosphatidylinositol3-kinase
pIgR: Polymeric immunoglobulin receptor
PRR: Pattern recognition receptor
RA: Rheumatoid arthritis
REGIIIγ: Regenerating islet-derived protein III-γ
SAP: Serum
SCFA: Short chain fatty acid
SLE: Systemic lupus erythematosus
SNP: Single nucleotide polymorphism
SYK: Spleen tyrosine kinase
TLR: Toll-like receptor
Th: T helper cell
TNF: Tumor necrosis factor
Treg: Regulatory T cell
TRIM21: Tripartite motif-containing protein 21
UC: Ulcerative colitis.

## References

[1] Cheng LK O'Grady G Du P Egbuji JU Windsor JA Pullan AJ. Gastrointestinal system. Wiley Interdiscipl Rev. (2010) 2:65–79. 10.1002/wsbm.19 20836011 PMC4221587

[2] Macpherson AJ Harris NL. Interactions between commensal intestinal bacteria and the immune system. Nat Rev Immunol. (2004) 4:478–85. 10.1038/nri1373 15173836

[3] Hooper LV Littman DR Macpherson AJ. Interactions between the microbiota and the immune system. Science. (2012) 336:1268–73. 10.1126/science.1223490 22674334 PMC4420145

[4] Hooper LV Macpherson AJ. Immune adaptations that maintain homeostasis with the intestinal microbiota. Nat Rev Immunol. (2010) 10:159–69. 10.1038/nri2710 20182457

[5] Gomez de Agüero M Ganal-Vonarburg SC Fuhrer T Rupp S Uchimura Y Li H The maternal microbiota drives early postnatal innate immune development. Science. (2016) 351:1296–302. 10.1126/science.aad2571 26989247

[6] Kamada N Sakamoto K Seo S-U Zeng MY Kim Y-G Cascalho M. Humoral immunity in the gut selectively targets phenotypically virulent attaching-and-effacing bacteria for intraluminal elimination. Cell Host Microbe. (2015) 17:617–27. 10.1016/j.chom.2015.04.001 25936799 PMC4433422

[7] Koch MA Reiner GL Lugo KA Kreuk LSM Stanbery AG Ansaldo E. Maternal IgG and IgA antibodies dampen mucosal T helper cell responses in early life. Cell. (2016) 165:827–41. 10.1016/j.cell.2016.04.055 27153495 PMC4866587

[8] Ohsaki A Venturelli N Buccigrosso TM Osganian SK Lee J Blumberg RS. Maternal IgG immune complexes induce food allergen-specific tolerance in offspring. J Exp Med. (2017) 215:91. 10.1084/jem.20171163 29158374 PMC5748859

[9] Baklien K Brandtzaeg P. Comparative mapping of the local distribution of immunoglobulin-containing cells in ulcerative colitis and Crohn's disease of the colon. Clin Exp Immunol. (1975) 22:197–209. 1082398 PMC1538296

[10] Benckert J Schmolka N Kreschel C Zoller MJ Sturm A Wiedenmann B. The majority of intestinal IgA + and IgG + plasmablasts in the human gut are antigen-specific. J Clin Invest. (2011) 121:1946–55. 10.1172/JCI44447 21490392 PMC3083800

[11] Lodes MJ Cong Y Elson CO Mohamath R Landers CJ Targan SR. Bacterial flagellin is a dominant antigen in Crohn disease. J Clin Invest. (2004) 113:1296–306. 10.1172/JCI200420295 15124021 PMC398429

[12] Anderson CA Boucher G Lees CW Franke A D′Amato M Taylor KD. Meta-analysis identifies 29 additional ulcerative colitis risk loci, increasing the number of confirmed associations to 47. Nat Genet. (2011) 43:246–52. 10.1038/ng.764 21297633 PMC3084597

[13] Asano K Matsushita T Umeno J Hosono N Takahashi A Kawaguchi T. A genome-wide association study identifies three new susceptibility loci for ulcerative colitis in the Japanese population. Nat Genet. (2009) 41:1325–9. 10.1038/ng.482 19915573

[14] Jostins L Ripke S Weersma RK Duerr RH McGovern DP Hui KY. Host-microbe interactions have shaped the genetic architecture of inflammatory bowel disease. Nature. (2012) 491:119–24. 10.1038/nature11582 23128233 PMC3491803

[15] Vidarsson G Dekkers G Rispens T. IgG subclasses and allotypes: from structure to effector functions. Front Immunol. (2014) 5:520. 10.3389/fimmu.2014.00520 25368619 PMC4202688

[16] Noris M Remuzzi G. Overview of complement activation and regulation. Semin Nephrol. (2013) 33:479–92. 10.1016/j.semnephrol.2013.08.001 24161035 PMC3820029

[17] Nimmerjahn F Ravetch JV. Fcgamma receptors as regulators of immune responses. Nat Rev Immunol. (2008) 8:34–47. 10.1038/nri2206 18064051

[18] Smith KGC Clatworthy MR. FcgammaRIIB in autoimmunity and infection: evolutionary and therapeutic implications. Nat Rev Immunol. (2010) 10:328–43. 10.1038/nri2762 20414206 PMC4148599

[19] Espéli M Smith KGC Clatworthy MR. FcγRIIB and autoimmunity. Immunol Rev. (2016) 269:194–211. 10.1111/imr.12368 26683154

[20] Bournazos S DiLillo DJ Ravetch JV The role of Fc-Fc R interactions in IgG-mediated microbial neutralization. J Exp Med. (2015) 212:1361–9. 10.1084/jem.20151267 26282878 PMC4548051

[21] Mallery DL McEwan WA Bidgood SR Towers GJ Johnson CM James LC. Antibodies mediate intracellular immunity through tripartite motif-containing 21 (TRIM21). Proc Natl Acad Sci USA. (2010) 107:19985–90. 10.1073/pnas.1014074107 21045130 PMC2993423

[22] Rath T Baker K Pyzik M Blumberg RS. Regulation of immune responses by the neonatal Fc receptor and its therapeutic implications. Front Immunol. (2014) 5:664. 10.3389/fimmu.2014.00664 25601863 PMC4283642

[23] Junghans RP Anderson CL. The protection receptor for IgG catabolism is the beta2-microglobulin-containing neonatal intestinal transport receptor. Proc Natl Acad Sc USA. (1993) 93:5512–6. 10.1073/pnas.93.11.5512 8643606 PMC39277

[24] Pyzik M Rath T Lencer WI Baker K Blumberg RS. FcRn: the architect behind the immune and nonimmune functions of IgG and Albumin. J Immunol. (2015) 194:4595–603. 10.4049/jimmunol.1403014 25934922 PMC4451002

[25] Yoshida M Claypool SM Wagner JS Mizoguchi E Mizoguchi A Roopenian DC. Human neonatal Fc receptor mediates transport of IgG into luminal secretions for delivery of antigens to mucosal dendritic cells. Immunity. (2004) 20:769–83. 10.1016/j.immuni.2004.05.007 15189741

[26] Yoshida M Kobayashi K Kuo TT Bry L Glickman JN Claypool SM. Neonatal Fc receptor for IgG regulates mucosal immune responses to luminal bacteria. J Clin Invest. (2006) 116:2142–51. 10.1172/JCI27821 16841095 PMC1501111

[27] Bharadwaj BD Stein M-P Volzer M Mold C Clos TWD The major receptor for C-reactive protein on leukocytes is Fcg receptor II. J Exp Med. (1999) 190:585–90. 10.1084/jem.190.4.585 10449529 PMC2195602

[28] Bharadwaj D Mold C Markham E Du Clos TW. Serum amyloid P component binds to Fc gamma receptors and opsonizes particles for phagocytosis. J Immunol. (2001) 166:6735–41. 10.4049/jimmunol.166.11.6735 11359830

[29] Pepys MB Booth SE Tennent GA Butler PJG Williams DG. Binding of pentraxins to different nuclear structures: C-reactive protein binds to small nuclear ribonucleoprotein particles, serum amyloid P component binds to chromatin and nucleoli. Clin Exp Immunol. (1994) 97:152–7. 10.1111/j.1365-2249.1994.tb06594.x 8033412 PMC1534802

[30] Vermeire S Van Assche G Rutgeerts P. The role of C-reactive protein as an inflammatory marker in gastrointestinal diseases. Nat Clin Pract Gastroenterol Hepatol. (2005) 2:580–6. 10.1038/ncpgasthep0359 16327837

[31] Bruhns P Iannascoli B England P Mancardi DA Fernandez N Jorieux S. Specificity and affinity of human Fcgamma receptors and their polymorphic variants for human IgG subclasses. Blood. (2009) 113:3716–25. 10.1182/blood-2008-09-179754 19018092

[32] Irani V Guy AJ Andrew D Beeson JG Ramsland PA Richards JS. Molecular properties of human IgG subclasses and their implications for designing therapeutic monoclonal antibodies against infectious diseases. Mol Immunol. (2015) 67:171–82. 10.1016/j.molimm.2015.03.255 25900877

[33] von Gunten S Smith DF Cummings RD Riedel S Miescher S Schaub A Intravenous immunoglobulin contains a broad repertoire of anticarbohydrate antibodies that is not restricted to the IgG2 subclass. J Allergy Clin Immunol. (2009) 123:1–16. 10.1016/j.jaci.2009.03.013 PMC2777748 19443021

[34] Stapleton NM Andersen JT Stemerding AM Bjarnarson SP Verheul RC Gerritsen J. Competition for FcRn-mediated transport gives rise to short half-life of human IgG3 and offers therapeutic potential. Nat Commun. (2011) 2:599–599. 10.1038/ncomms1608 22186895 PMC3247843

[35] van der Neut Kolfschoten M Schuurman J Losen M Bleeker WK Martínez-Martínez P Vermeulen E Anti-inflammatory activity of human IgG4 antibodies by dynamic Fab arm exchange. Science. (2007) 317:1554–7. 10.1126/science.1144603 17872445

[36] Aalberse RC Schuurman J. IgG4 breaking the rules. Immunology. (2002) 105:9–19. 10.1046/j.0019-2805.2001.01341.x 11849310 PMC1782638

[37] Kaneko Y Nimmerjahn F Ravetch JV. Anti-inflammatory activity of immunoglobulin G resulting from Fc sialylation. Science. (2006) 313:670–3. 10.1126/science.1129594 16888140

[38] Karsten CM Pandey MK Figge J Kilchenstein R Taylor PR Rosas M. Anti-inflammatory activity of IgG1 mediated by Fc galactosylation and association of FcγRIIB and dectin-1. Nat Med. (2012) 18:1401–6. 10.1038/nm.2862 22922409 PMC3492054

[39] Li T DiLillo DJ Bournazos S Giddens JP Ravetch JV Wang L-X. Modulating IgG effector function by Fc glycan engineering. Proc Natl Acad Sci USA. (2017) 114:3485–90. 10.1073/pnas.1702173114 28289219 PMC5380036

[40] Pincetic A Bournazos S DiLillo DJ Maamary J Wang TT Dahan R. Type I and type II Fc receptors regulate innate and adaptive immunity. Nat Immunol. (2014) 15:707–16. 10.1038/ni.2939 25045879 PMC7430760

[41] Lauc G Huffman JE Pučić M Zgaga L Adamczyk B MuŽinić A. Loci associated with N-glycosylation of human immunoglobulin G show pleiotropy with autoimmune diseases and haematological cancers. PLoS Genet. (2013) 9:e1003225. 10.1371/journal.pgen.1003225 23382691 PMC3561084

[42] Theodoratou E Campbell H Ventham NT Kolarich D Pučić-Baković M Zoldoš V. The role of glycosylation in IBD. Nat Rev Gastroenterol Hepatol. (2014) 11:588–600. 10.1038/nrgastro.2014.78 24912389

[43] Arnold JN Wormald MR Sim RB Rudd PM Dwek RA. The impact of glycosylation on the biological function and structure of human immunoglobulins. Annu Rev Immunol. (2007) 25:21–50. 10.1146/annurev.immunol.25.022106.141702 17029568

[44] Anthony RM Ravetch JV. A novel role for the IgG Fc glycan: the anti-inflammatory activity of sialylated IgG Fcs. J Clin Immunol. (2010) 30:9–14. 10.1007/s10875-010-9405-6 20480216

[45] Anthony RM Wermeling F Karlsson MCI Ravetch JV. Identification of a receptor required for the anti-inflammatory activity of IVIG. Proc Natl Acad Sci USA. (2008) 105:19571–8. 10.1073/pnas.0810163105 19036920 PMC2604916

[46] Dong X Storkus WJ Salter RD. Binding and uptake of agalactosyl IgG by mannose receptor on macrophages and dendritic cells. J Immunol. (1999) 163:5427–34. 10553068

[47] Malhotra R Wormald MR Rudd PM Fischer PB Dwek RA Sim RB. Glycosylation changes of IgG associated with rheumatoid arthritis can activate complement via the mannose-binding protein. Nat Med. (1995) 1:237–43. 10.1038/nm0395-237 7585040

[48] McGuckin MA Eri R Simms LA Florin THJ Radford-Smith G. Intestinal barrier dysfunction in inflammatory bowel diseases. Inflamm Bowel Dis. (2009) 15:100–13. 10.1002/ibd.20539 18623167

[49] Peterson LW Artis D. Intestinal epithelial cells: regulators of barrier function and immune homeostasis. Nat Rev Immunol. (2014) 14:141–53. 10.1038/nri3608 24566914

[50] Elinav E Strowig T Kau AL Henao-Mejia J Thaiss CA Booth CJ. NLRP6 inflammasome regulates colonic microbial ecology and risk for colitis. Cell. (2011) 145:745–57. 10.1016/j.cell.2011.04.022 21565393 PMC3140910

[51] Fulde M Sommer F Chassaing B van Vorst K Dupont A Hensel M. Neonatal selection by Toll-like receptor 5 influences long-term gut microbiota composition. Nature. (2018) 560:489–93. 10.1038/s41586-018-0395-5 30089902

[52] Price AE Shamardani K Lugo KA Deguine J Roberts AW Lee BL. A map of toll-like receptor expression in the intestinal epithelium reveals distinct spatial, cell type-specific, and temporal patterns. Immunity. (2018) 49:560–75.e6. 10.1016/j.immuni.2018.07.016 30170812 PMC6152941

[53] Harrison OJ Srinivasan N Pott J Schiering C Krausgruber T Ilott NE. Epithelial-derived IL-18 regulates Th17 cell differentiation and Foxp3+ Treg cell function in the intestine. Mucosal Immunol. (2015) 8:1226–36. 10.1038/mi.2015.13 25736457 PMC4368110

[54] Nowarski R Jackson R Gagliani N de Zoete MR Palm NW Bailis W. Epithelial IL-18 equilibrium controls barrier function in colitis. Cell. (2015) 163:1444–56. 10.1016/j.cell.2015.10.072 26638073 PMC4943028

[55] Sawa S Lochner M Satoh-Takayama N Dulauroy S Bérard M Kleinschek M. RORγt+ innate lymphoid cells regulate intestinal homeostasis by integrating negative signals from the symbiotic microbiota. Nature immunology. (2011) 12:320–6. 10.1038/ni.2002 21336274

[56] Rimoldi M Chieppa M Salucci V Avogadri F Sonzogni A Sampietro GM. Intestinal immune homeostasis is regulated by the crosstalk between epithelial cells and dendritic cells. Nat Immunol. (2005) 6:507–14. 10.1038/ni1192 15821737

[57] He B Xu W Santini PA Polydorides AD Chiu A Estrella J Intestinal bacteria trigger T cell-independent Immunoglobulin A2 class switching by inducing epithelial-cell secretion of the cytokine APRIL. Immunity. (2007) 26:812–26. 10.1016/j.immuni.2007.04.014 17570691

[58] Cho JH Brant SR. Recent insights into the genetics of inflammatory bowel disease. Gastroenterology. (2011) 140:1704–12. 10.1053/j.gastro.2011.02.046 21530736 PMC4947143

[59] Mu Q Kirby J Reilly CM Luo XM. Leaky gut as a danger signal for autoimmune diseases. Front Immunol. (2017) 8:598. 10.3389/fimmu.2017.00598 28588585 PMC5440529

[60] Coccia M Harrison OJ Schiering C Asquith MJ Becher B Powrie F. IL-1β mediates chronic intestinal inflammation by promoting the accumulation of IL-17A secreting innate lymphoid cells and CD4(+) Th17 cells. J Exp Med. (2012) 209:1595–609. 10.1084/jem.20111453 22891275 PMC3428945

[61] Duerr RH Taylor KD Brant SR Rioux JD Silverberg MS Daly MJ. A genome-wide association study identifies IL23R as an inflammatory bowel disease gene. Science. (2006) 314:1461–3. 10.1126/science.1135245 17068223 PMC4410764

[62] Ivanov II Atarashi K Manel N Brodie EL Shima T Karaoz U. Induction of intestinal Th17 cells by segmented filamentous bacteria. Cell. (2009) 139:485–98. 10.1016/j.cell.2009.09.033 19836068 PMC2796826

[63] Neurath MF. Cytokines in inflammatory bowel disease. Nat Rev Immunol. (2014) 14:329–42. 10.1038/nri3661 24751956

[64] Sano T Huang W Hall JA Yang Y Chen A Gavzy SJ. An IL-23R/IL-22 circuit regulates epithelial serum amyloid a to promote local effector Th17 responses. Cell. (2015) 163:381–93. 10.1016/j.cell.2015.08.061 26411290 PMC4621768

[65] Withers DR Hepworth MR Wang X Mackley EC Halford EE Dutton EE. Transient inhibition of ROR-γt therapeutically limits intestinal inflammation by reducing TH17 cells and preserving group 3 innate lymphoid cells. Nat Med. (2016) 22:319–23. 10.1038/nm.4046 26878233 PMC4948756

[66] Jovanovic DV Di Battista JA Martel-Pelletier J Jolicoeur FC He Y Zhang M. IL-17 stimulates the production and expression of proinflammatory cytokines, IL-beta and TNF-alpha, by human macrophages. J Immunol. (1998) 160:3513–21. 9531313

[67] Korn T Bettelli E Oukka M Kuchroo VK. IL-17 and Th17 Cells. Annu Rev Immunol. (2009) 27:485–517. 10.1146/annurev.immunol.021908.132710 19132915

[68] Veldhoen M. Interleukin 17 is a chief orchestrator of immunity. Nat Immunol. (2017) 18:612–21. 10.1038/ni.3742 28518156

[69] Xiong H Keith JW Samilo DW Carter RA Leiner IM Pamer EG. Innate Lymphocyte/Ly6C(hi) monocyte crosstalk promotes klebsiella pneumoniae clearance. Cell. (2016) 165:679–89. 10.1016/j.cell.2016.03.017 27040495 PMC4842125

[70] Laan M Cui ZH Hoshino H Lötvall J Sjöstrand M Gruenert DC. Neutrophil recruitment by human IL-17 via C-X-C chemokine release in the airways. J Immunol. (1999) 162:2347–52. 9973514

[71] Parsonage G Filer A Bik M Hardie D Lax S Howlett K. Prolonged, granulocyte-macrophage colony-stimulating factor-dependent, neutrophil survival following rheumatoid synovial fibroblast activation by IL-17 and TNFalpha. Arthritis Res Ther. (2008) 10:R47–R47. 10.1186/ar2406 18433499 PMC2453767

[72] Witowski J Pawlaczyk K Breborowicz A Scheuren A Kuzlan-Pawlaczyk M Wisniewska J. IL-17 stimulates intraperitoneal neutrophil infiltration through the release of GRO chemokine from mesothelial cells. J Immunol. (2000) 165:5814–21. 10.4049/jimmunol.165.10.5814 11067941

[73] Kolls JK McCray PB Chan YR. Cytokine-mediated regulation of antimicrobial proteins. Nat Rev Immunol. (2008) 8:829–35. 10.1038/nri2433 18949018 PMC2901862

[74] Lee JS Tato CM Joyce-Shaikh B Gulan F Cayatte C Chen Y. Interleukin-23-independent IL-17 production regulates intestinal epithelial permeability. Immunity. (2015) 43:727–38. 10.1016/j.immuni.2015.09.003 26431948 PMC6044435

[75] Maxwell JR Zhang Y Brown WA Smith CL Byrne FR Fiorino M. Differential Roles for Interleukin-23 and Interleukin-17 in intestinal immunoregulation. Immunity. (2015) 43:739–50. 10.1016/j.immuni.2015.08.019 26431947

[76] Song X Dai D He X Zhu S Yao Y Gao H. Growth factor FGF2 cooperates with Interleukin-17 to repair intestinal epithelial damage. Immunity. (2015) 43:488–501. 10.1016/j.immuni.2015.06.024 26320657

[77] Kamada N Hisamatsu T Okamoto S Chinen H Kobayashi T Sato T. Unique CD14 intestinal macrophages contribute to the pathogenesis of Crohn disease via IL-23/IFN-gamma axis. J Clin Invest. (2008) 118:2269–80. 10.1172/JCI34610 18497880 PMC2391067

[78] Ito R Shin-Ya M Kishida T Urano A Takada R Sakagami J. Interferon-gamma is causatively involved in experimental inflammatory bowel disease in mice. Clin Exp Immunol. (2006) 146:330–8. 10.1111/j.1365-2249.2006.03214.x 17034586 PMC1942055

[79] Neurath MF Weigmann B Finotto S Glickman J Nieuwenhuis E Iijima H. The transcription factor T-bet regulates mucosal T cell activation in experimental colitis and Crohn's disease. J Exp Med. (2002) 195:1129–43. 10.1084/jem.20011956 11994418 PMC2193714

[80] Powrie F Leach MW Mauze S Menon S Barcomb Caddle L Coffman RL Inhibition of Thl responses prevents inflammatory bowel disease in scid mice reconstituted with CD45RBhi CD4+ T cells. Immunity. (1994) 1:553–62. 10.1016/1074-7613(94)90045-0 7600284

[81] Barrett JC Lee JC Lees CW Prescott NJ Anderson CA Phillips A. Genome-wide association study of ulcerative colitis identifies three new susceptibility loci, including the HNF4A region. Nat Genet. (2009) 41:1330–4. 10.1038/ng.483 19915572 PMC2812019

[82] Fox JG Ge Z Whary MT Erdman SE Horwitz BH. Helicobacter hepaticus infection in mice: models for understanding lower bowel inflammation and cancer. Mucosal Immunol. (2011) 4:22–30. 10.1038/mi.2010.61 20944559 PMC3939708

[83] Glocker E-O Kotlarz D Boztug K Gertz EM Schäffer AA Noyan F. Inflammatory bowel disease and mutations affecting the interleukin-10 receptor. N Engl J Med. (2009) 361:2033–45. 10.1056/NEJMoa0907206 19890111 PMC2787406

[84] Olszak T Neves JF Dowds CM Baker K Glickman J Davidson NO. Protective mucosal immunity mediated by epithelial CD1d and IL-10. Nature. (2014) 509:497–502. 10.1038/nature13150 24717441 PMC4132962

[85] Rosser EC Oleinika K Tonon S Doyle R Bosma A Carter NA. Regulatory B cells are induced by gut microbiota-driven interleukin-1β and interleukin-6 production. Nat Med. (2014) 20:1334–9. 10.1038/nm.3680 25326801

[86] Sattler S Ling GS Xu D Hussaarts L Romaine A Zhao H. IL-10-producing regulatory B cells induced by IL-33 (BregIL-33) effectively attenuate mucosal inflammatory responses in the gut. Jf Autoimmun. (2014) 50:107–22. 10.1016/j.jaut.2014.01.032 24491821 PMC4012142

[87] Pabst O. New concepts in the generation and functions of IgA. Nat Rev Immunol. (2012) 12:821–32. 10.1038/nri3322 23103985

[88] Cerutti A. The regulation of IgA class switching. Nat Rev Immunol. (2008) 8:421–34. 10.1038/nri2322 18483500 PMC3062538

[89] Bollinger RR Everett ML Wahl SD Lee YH Orndorff PE Parker W. Secretory IgA and mucin-mediated biofilm formation by environmental strains of Escherichia coli: role of type 1 pili. Mol Immunol. (2006) 43:378–87. 10.1016/j.molimm.2005.02.013 16310051

[90] Moor K Diard M Sellin ME Felmy B Wotzka SY Toska A. High-avidity IgA protects the intestine by enchaining growing bacteria. Nature. (2017) 544:498–502. 10.1038/nature22058 28405025

[91] Fransen F Zagato E Mazzini E Fosso B Manzari C El Aidy S. BALB/c and C57BL/6 mice differ in polyreactive IgA abundance, which impacts the generation of antigen-specific IgA and microbiota diversity. Immunity. (2015) 43:527–40. 10.1016/j.immuni.2015.08.011 26362264

[92] Lécuyer E Rakotobe S Lengliné-Garnier H Lebreton C Picard M Juste C. Segmented filamentous bacterium uses secondary and tertiary lymphoid tissues to induce gut IgA and specific T helper 17 cell responses. Immunity. (2014) 40:608–20. 10.1016/j.immuni.2014.03.009 24745335

[93] Tsuji M Suzuki K Kitamura H Maruya M Kinoshita K Ivanov II. Requirement for lymphoid tissue-inducer cells in isolated follicle formation and T cell-independent Immunoglobulin A generation in the gut. Immunity. (2008) 29:261–71. 10.1016/j.immuni.2008.05.014 18656387

[94] Masahata K Umemoto E Kayama H Kotani M Nakamura S Kurakawa T. Generation of colonic IgA-secreting cells in the caecal patch. Nat Commun. (2014) 5:3704–3704. 10.1038/ncomms4704 24718324

[95] Milpied PJ McHeyzer-Williams MG. High-affinity IgA needs TH17 cell functional plasticity. Nat Immunol. (2013) 14:313–5. 10.1038/ni.2567 23507637

[96] Mora JR Iwata M Eksteen B Song S-Y Junt T Senman B. Generation of gut-homing IgA-secreting B cells by intestinal dendritic cells. Science. (2006) 314:1157–60. 10.1126/science.1132742 17110582

[97] Reboldi A Arnon TI Rodda LB Atakilit A Sheppard D Cyster JG. IgA production requires B cell interaction with subepithelial dendritic cells in Peyer's patches. Science. (2016) 352:1–10. 10.1126/science.aaf4822 27174992 PMC4890166

[98] Brandtzaeg P Johansen F-E. Mucosal B cells: phenotypic characteristics, transcriptional regulation, and homing properties. Immunol Rev. (2005) 206:32–63. 10.1111/j.0105-2896.2005.00283.x 16048541

[99] Pone EJ Zan H Zhang J Al-Qahtani A Xu Z Casali P. Toll-like receptors and B-cell receptors synergize to induce immunoglobulin class-switch DNA recombination: relevance to microbial antibody responses. Crit Rev Immunol. (2010) 30:1–29. 10.1615/CritRevImmunol.v30.i1.10 20370617 PMC3038989

[100] Pone EJ Zhang J Mai T White CA Li G Sakakura JK. BCR-signalling synergizes with TLR-signalling for induction of AID and immunoglobulin class-switching through the non-canonical NF-κB pathway. Nat Commun. (2012) 3:767–767. 10.1038/ncomms1769 22473011 PMC3337981

[101] Moor K Wotzka SY Toska A Diard M Hapfelmeier S Slack E. Peracetic acid treatment generates potent inactivated oral vaccines from a broad range of culturable bacterial species. Front Immunol. (2016) 7:34. 10.3389/fimmu.2016.00034 26904024 PMC4749699

[102] Maaser C Housley MP Iimura M Smith JR Vallance BA Finlay BB Clearance of *Citrobacter rodentium* requires B cells but not secretory immunoglobulin A (IgA) or IgM antibodies. Infect Immun. (2004) 72:3315–24. 10.1128/IAI.72.6.3315-3324.2004 15155635 PMC415672

[103] Panda S Zhang J Tan NS Ho B Ding JL. Natural IgG antibodies provide innate protection against ficolin-opsonized bacteria. EMBO J. (2013) 32:2905–19. 10.1038/emboj.2013.199 24002211 PMC3831310

[104] Gabanyi I Muller PA Feighery L Oliveira TY Costa-Pinto FA Mucida D. Neuro-immune interactions drive tissue programming in intestinal macrophages. Cell. (2016) 164:378–91. 10.1016/j.cell.2015.12.023 26777404 PMC4733406

[105] Gross M Salame T-M Jung S. Guardians of the gut – murine intestinal macrophages and dendritic cells. Front Immunol. (2015) 6:254. 10.3389/fimmu.2015.00254 26082775 PMC4451680

[106] Muller PA Koscsó B Rajani GM Stevanovic K Berres ML Hashimoto D Crosstalk between muscularis macrophages and enteric neurons regulates gastrointestinal motility. Cell. (2014) 158:300–13. 10.1016/j.cell.2014.04.050 25036630 PMC4149228

[107] Bain CC Bravo-Blas A Scott CL Perdiguero EG Geissmann F Henri S. Constant replenishment from circulating monocytes maintains the macrophage pool in the intestine of adult mice. Nat Immunol. (2014) 15:929–37. 10.1038/ni.2967 25151491 PMC4169290

[108] Kuhn KA Stappenbeck TS. Peripheral education of the immune system by the colonic microbiota. Semin Immunol. (2013) 25:364–9. 10.1016/j.smim.2013.10.002 24169518 PMC3864652

[109] Thaiss CA Zmora N Levy M Elinav E. The microbiome and innate immunity. Nature. (2016) 535:65–74. 10.1038/nature18847 27383981

[110] Chang PV Hao L Offermanns S Medzhitov R. The microbial metabolite butyrate regulates intestinal macrophage function via histone deacetylase inhibition. Proc Natl Acad Sci USA. (2014) 111:2247–52. 10.1073/pnas.1322269111 24390544 PMC3926023

[111] Krause P Morris V Greenbaum JA Park Y Bjoerheden U Mikulski Z. IL-10-producing intestinal macrophages prevent excessive antibacterial innate immunity by limiting IL-23 synthesis. Nat Commun. (2015) 6:1–12. 10.1038/ncomms8055 25959063 PMC4428691

[112] Zigmond E Varol C Farache J Elmaliah E Satpathy AT Friedlander G Ly6Chi monocytes in the inflamed colon give rise to proinflammatory effector cells and migratory antigen-presenting cells. Immunity. (2012) 37:1076–90. 10.1016/j.immuni.2012.08.026 23219392

[113] Denning TL Wang Y-C Patel SR Williams IR Pulendran B. Lamina propria macrophages and dendritic cells differentially induce regulatory and interleukin 17-producing T cell responses. Nat Immunol. (2007) 8:1086–94. 10.1038/ni1511 17873879

[114] Bain CC Scott CL Uronen-Hansson H Gudjonsson S Jansson O Grip O. Resident and pro-inflammatory macrophages in the colon represent alternative context-dependent fates of the same Ly6Chi monocyte precursors. Mucosal Immunol. 6:498–510. 10.1038/mi.2012.89 22990622 PMC3629381

[115] Arnold IC Mathisen S Schulthess J Danne C Hegazy AN Powrie F. CD11c+ monocyte/macrophages promote chronic Helicobacter hepaticus-induced intestinal inflammation through the production of IL-23. Mucosal Immunol. (2015) 9: 352–63. 10.1038/mi.2015.65 26242598 PMC4650208

[116] Asano K Takahashi N Ushiki M Monya M Aihara F Kuboki E. Intestinal CD169+ macrophages initiate mucosal inflammation by secreting CCL8 that recruits inflammatory monocytes. Nat Commun. (2015) 6:7802. 10.1038/ncomms8802 26193821 PMC4518321

[117] Buonocore S Ahern PP Uhlig HH Ivanov II Littman DR Maloy KJ. Innate lymphoid cells drive interleukin-23-dependent innate intestinal pathology. Nature. (2010) 464:1371–5. 10.1038/nature08949 20393462 PMC3796764

[118] Longman RS Diehl GE Victorio DA Huh JR Galan C Miraldi ER CX3CR1+ mononuclear phagocytes support colitis-associated innate lymphoid cell production of IL-22. J Exp Med. (2014) 211:1571 10.1084/jem.20140678 25024136 PMC4113938

[119] Seo S-U Kamada N Muñoz-Planillo R Kim Y-G Kim D Koizumi Y. Distinct commensals induce Interleukin-1β via NLRP3 inflammasome in inflammatory monocytes to promote intestinal inflammation in response to injury. Immunity. (2015) 42:744–55. 10.1016/j.immuni.2015.03.004 25862092 PMC4408263

[120] Varol C Mildner A Jung S. Macrophages: development and tissue specialization. Annu Rev Immunol. (2015) 33:643–75. 10.1146/annurev-immunol-032414-112220 25861979

[121] Tamoutounour S Henri S Lelouard H de Bovis B de Haar C van der Woude CJ. CD64 distinguishes macrophages from dendritic cells in the gut and reveals the Th1-inducing role of mesenteric lymph node macrophages during colitis. Eur J Immunol. (2012) 42:3150–66. 10.1002/eji.201242847 22936024

[122] Anthony RM Kobayashi T Wermeling F Ravetch JV. Intravenous gammaglobulin suppresses inflammation through a novel T(H)2 pathway. Nature. (2011) 475:110–3. 10.1038/nature10134 21685887 PMC3694429

[123] Pricop L Redecha P Teillaud JL Frey J Fridman WH Sautes-Fridman C. Differential modulation of stimulatory and inhibitory Fc receptors on human monocytes by Th1 and Th2 cytokines. J Immunol. (2001) 166:531–7. 10.4049/jimmunol.166.1.531 11123333

[124] Samuelsson A Towers TL Ravetch JV. Anti-inflammatory activity of IVIG mediated through the inhibitory Fc receptor. Science. (2001) 291:484–6. 10.1126/science.291.5503.484 11161202

[125] Bournazos S Wang TT Ravetch JV. The role and function of Fcγ receptors on myeloid cells. Microbiol Spectr. (2016) 4:1–19. 10.1128/microbiolspec.MCHD-0045-2016 28087938 PMC5240797

[126] Guilliams M Bruhns P Saeys Y Hammad H Lambrecht BN. The function of Fcγ receptors in dendritic cells and macrophages. Nat Rev Immunol. (2014) 14:94–108. 10.1038/nri3582 24445665

[127] Dhodapkar KM Banerjee D Connolly J Kukreja A Matayeva E Veri MC Selective blockade of the inhibitory Fcg receptor (FcgRIIB) in human dendritic cells and monocytes induces a type I interferon response program. J Exp Med. (2007) 204:1359–69. 10.1084/jem.20062545 17502666 PMC2118610

[128] Bakema JE Tuk CW van Vliet SJ Bruijns SC Vos JB Letsiou S. Antibody-opsonized bacteria evoke an inflammatory dendritic cell phenotype and polyfunctional Th cells by cross-talk between TLRs and FcRs. J Immunol. (2015) 194:1856–66. 10.4049/jimmunol.1303126 25582855

[129] den Dunnen J Vogelpoel LTC Wypych T Muller FJM de Boer L Kuijpers TW IgG opsonization of bacteria promotes Th17 responses via synergy between TLRs and FcγRIIa in human dendritic cells. Blood. (2012) 120:112–21. 10.1182/blood-2011-12-399931 22649103

[130] Vogelpoel LTC Hansen IS Rispens T Muller FJM van Capel TMM Turina MC. Fc gamma receptor-TLR cross-talk elicits pro-inflammatory cytokine production by human M2 macrophages. Nat Commun. (2014) 5:5444–5444. 10.1038/ncomms6444 25392121 PMC4243215

[131] Janczy JR Ciraci C Haasken S Iwakura Y Olivier AK Cassel SL. Immune complexes inhibit IL-1 secretion and inflammasome activation. J Immunol. (2014) 193:5190–8. 10.4049/jimmunol.1400628 25320279 PMC4225162

[132] Zhang Y Liu S Liu J Zhang T Shen Q Yu Y. Immune complex/Ig negatively regulate TLR4-triggered inflammatory response in macrophages through Fc gamma RIIb-dependent PGE2 production. J Immunol. (2009) 182:554–62. 10.4049/jimmunol.182.1.554 19109188

[133] Coombes JL Powrie F. Dendritic cells in intestinal immune regulation. Nat Rev Immunol. (2008) 8:435–46. 10.1038/nri2335 18500229 PMC2674208

[134] Guilliams M Ginhoux F Jakubzick C Naik SH Onai N Schraml BU. Dendritic cells, monocytes and macrophages: a unified nomenclature based on ontogeny. Nat Rev Immunol. (2014) 14:571–8. 10.1038/nri3712 25033907 PMC4638219

[135] Persson E Uronen-Hansson H Semmrich M Rivollier A Hägerbrand K Marsal J. IRF4 transcription-factor-dependent CD103+CD11b+ dendritic cells drive mucosal T helper 17 cell differentiation. Immunity. (2013) 38:958–69. 10.1016/j.immuni.2013.03.009 23664832

[136] Flores-Langarica A Müller Luda K Persson EK Cook CN Bobat S Marshall JL. CD103+CD11b+ mucosal classical dendritic cells initiate long-term switched antibody responses to flagellin. Mucosal Immunol. (2017) 11:681–92. 10.1038/mi.2017.105 29346347 PMC5912514

[137] Cerovic V Houston SA Scott CL Aumeunier A Yrlid U Mowat AM. Intestinal CD103(-) dendritic cells migrate in lymph and prime effector T cells. Mucosal Immunol. (2013) 6:104–13. 10.1038/mi.2012.53 22718260

[138] Scott CL Bain CC Wright PB Sichien D Kotarsky K Persson EK. CCR2+CD103– intestinal dendritic cells develop from DC-committed precursors and induce interleukin-17 production by T cells. Mucosal Immunol. (2015) 8:327–39. 10.1038/mi.2014.70 25138666 PMC4270738

[139] Kinnebrew MA Buffie CG Diehl GE Zenewicz LA Leiner I Hohl TM. Interleukin 23 production by intestinal CD103(+)CD11b(+) dendritic cells in response to bacterial flagellin enhances mucosal innate immune defense. Immunity. (2012) 36:276–87. 10.1016/j.immuni.2011.12.011 22306017 PMC3288454

[140] Mundy R MacDonald TT Dougan G Frankel G Wiles S. *Citrobacter rodentium* of mice and man. Cell. Microbiol. (2005) 7:1697–706. 10.1111/j.1462-5822.2005.00625.x 16309456

[141] Satpathy AT Briseño CG Lee JS Ng D Manieri NA Kc W. Notch2-dependent classical dendritic cells orchestrate intestinal immunity to attaching-and-effacing bacterial pathogens. Nat Immunol. (2013) 14:937–48. 10.1038/ni.2679 23913046 PMC3788683

[142] Coombes JL Siddiqui KRR Arancibia-Cárcamo CV Hall J Sun C-M Belkaid Y. A functionally specialized population of mucosal CD103+ DCs induces Foxp3+ regulatory T cells via a TGF-beta and retinoic acid-dependent mechanism. J Exp Med. (2007) 204:1757–64. 10.1084/jem.20070590 17620361 PMC2118683

[143] Boruchov AM Heller G Veri M-C Bonvini E Ravetch JV Young JW. Activating and inhibitory IgG Fc receptors on human DCs mediate opposing functions. J Clin Invest. (2005) 115:2914–23. 10.1172/JCI24772 16167082 PMC1201664

[144] Dhodapkar KM Kaufman JL Ehlers M Banerjee DK Bonvini E Koenig S. Selective blockade of inhibitory Fcgamma receptor enables human dendritic cell maturation with IL-12p70 production and immunity to antibody-coated tumor cells. Proc Natl. Acad Sci USA. (2005) 102:2910–5. 10.1073/pnas.0500014102 15703291 PMC549508

[145] De Jong JMH Schuurhuis DH Ioan-Facsinay A Welling MM Camps MGM Van Der Voort EIH. Dendritic cells, but not macrophages or B cells, activate major histocompatibility complex class II-restricted CD4+ T cells upon immune-complex uptake *in vivo*. Immunology. (2006) 119:499–506. 10.1111/j.1365-2567.2006.02464.x 16995881 PMC2265814

[146] Regnault A Lankar D Lacabanne V Rodriguez A Théry C Rescigno M. Fcgamma receptor-mediated induction of dendritic cell maturation and major histocompatibility complex class I-restricted antigen presentation after immune complex internalization. J Exp Med. (1999) 189:371–80. 10.1084/jem.189.2.371 9892619 PMC2192989

[147] van Montfoort N t Hoen PAC Mangsbo SM Camps MGM Boross P Melief CJM Fc receptor IIb strongly regulates Fc receptor-facilitated T cell activation by dendritic cells. J Immunol. (2012) 189:92–101. 10.4049/jimmunol.1103703 22649202

[148] Clatworthy MR Aronin CEP Mathews RJ Morgan NY Smith KGC Germain RN. Immune complexes stimulate CCR7-dependent dendritic cell migration to lymph nodes. Nat Med. (2014) 20:1458–63. 10.1038/nm.3709 25384086 PMC4283039

[149] Baker K Qiao SW Kuo TT Aveson VG Platzer B Andersen JT. Neonatal Fc receptor for IgG (FcRn) regulates cross-presentation of IgG immune complexes by CD8-CD11b+ dendritic cells. Proc Natl Acad Sci USA. (2011) 108:9927–32. 10.1073/pnas.1019037108 21628593 PMC3116387

[150] Ley K Hoffman HM Kubes P Cassatella MA Zychlinsky A Hedrick CC. Neutrophils: new insights and open questions. Sci Immunol. (2018) 3:eaat4579. 30530726 10.1126/sciimmunol.aat4579

[151] Puga I Cols M Barra CM He B Cassis L Gentile M. B cell-helper neutrophils stimulate the diversification and production of immunoglobulin in the marginal zone of the spleen. Nat Immunol. (2012) 13:170–80. 10.1038/ni.2194 22197976 PMC3262910

[152] Beauvillain C Delneste Y Scotet M Peres A Gascan H Guermonprez P. Neutrophils efficiently cross-prime naive T cells *in vivo*. Blood. (2007) 110:2965–73. 10.1182/blood-2006-12-063826 17562875

[153] Culshaw S Millington OR Brewer JM McInnes IB. Murine neutrophils present Class II restricted antigen. Immunol Lett. (2008) 118:49–54. 10.1016/j.imlet.2008.02.008 18400308 PMC2430030

[154] Vono M Lin A Norrby-Teglund A Koup RA Liang F Loré K. Neutrophils acquire the capacity for antigen presentation to memory CD4 + T cells *in vitro* and *ex vivo*. Blood. (2017) 129:1991–2001. 10.1182/blood-2016-10-744441 28143882 PMC5383872

[155] Fournier BM Parkos CA. The role of neutrophils during intestinal inflammation. Mucosal Immunol. (2012) 5:354–66. 10.1038/mi.2012.24 22491176

[156] Kaser A Zeissig S Blumberg RS. Inflammatory bowel disease. Annu Rev Immunol. (2010) 28:573–621. 10.1146/annurev-immunol-030409-101225 20192811 PMC4620040

[157] Broggi A Tan Y Granucci F Zanoni I. IFN-λ suppresses intestinal inflammation by non-translational regulation of neutrophil function. Nat Immunol. (2017) 18:1084–93. 10.1038/ni.3821 28846084 PMC5701513

[158] Buell MG Berin MC. Neutrophil-independence of the initiation of colonic injury. Digest Dis Sci. (1994) 39:2575–88. 10.1007/BF02087693 7995182

[159] Qualls JE Kaplan AM Van Rooijen N Cohen DA. Suppression of experimental colitis by intestinal mononuclear phagocytes. J Leukoc Biol. (2006) 80:802–15. 10.1189/jlb.1205734 16888083

[160] Zindl CL Lai JF Lee YK Maynard CL Harbour SN Ouyang W. IL-22-producing neutrophils contribute to antimicrobial defense and restitution of colonic epithelial integrity during colitis. Proc Natl Acad Sci USA. (2013) 110:12768–73. 10.1073/pnas.1300318110 23781104 PMC3732935

[161] Casanova-Acebes M Nicolás-Ávila JA Li JL García-Silva S Balachander A Rubio-Ponce A. Neutrophils instruct homeostatic and pathological states in naive tissues. J Exp Med. (2018) 215:2778. 10.1084/jem.20181468 30282719 PMC6219739

[162] Ericson JA Duffau P Yasuda K Ortiz-Lopez A Rothamel K Rifkin IR. Gene expression during the generation and activation of mouse neutrophils: implication of novel functional and regulatory pathways. PLoS ONE. (2014) 9:e108553. 10.1371/journal.pone.0108553 25279834 PMC4184787

[163] Nimmerjahn F Bruhns P Horiuchi K Ravetch JV. FcgammaRIV: a novel FcR with distinct IgG subclass specificity. Immunity. (2005) 23:41–51. 10.1016/j.immuni.2005.05.010 16039578

[164] Willcocks LC Lyons PA Clatworthy MR Robinson JI Yang W Newland SA. Copy number of FCGR3B, which is associated with systemic lupus erythematosus, correlates with protein expression and immune complex uptake. J Exp Med. (2008) 205:1573–82. 10.1084/jem.20072413 18559452 PMC2442635

[165] Sur Chowdhury C Giaglis S Walker UA Buser A Hahn S Hasler P Enhanced neutrophil extracellular trap generation in rheumatoid arthritis: analysis of underlying signal transduction pathways and potential diagnostic utility. Arthritis Res Ther. (2014) 16:R122 10.1186/ar4579 24928093 PMC4229860

[166] Kobayashi SD Voyich JM Buhl CL Stahl RM DeLeo FR. Global changes in gene expression by human polymorphonuclear leukocytes during receptor-mediated phagocytosis: cell fate is regulated at the level of gene expression. Proc Natl Acad Sci USA. (2002) 99:6901–6. 10.1073/pnas.092148299 11983860 PMC124501

[167] Sadik CD Kim ND Iwakura Y Luster AD. Neutrophils orchestrate their own recruitment in murine arthritis through C5aR and FcγR signaling. Proc Natl Acad Sci USA. (2012) 109:E3177–85. 10.1073/pnas.1213797109 23112187 PMC3503206

[168] Coxon A Cullere X Knight S Sethi S Wakelin MW Stavrakis G. Fc gamma RIII mediates neutrophil recruitment to immune complexes. a mechanism for neutrophil accumulation in immune-mediated inflammation. Immunity. (2001) 14:693–704. 10.1016/S1074-7613(01)00150-9 11420040

[169] Mayadas TN Tsokos GC Tsuboi N. Mechanisms of immune complex-mediated neutrophil recruitment and tissue injury. Circulation. (2009) 120:2012–24. 10.1161/CIRCULATIONAHA.108.771170 19917895 PMC2782878

[170] Zhou MJ Brown EJ. CR3 (Mac-1, alpha M beta 2, CD11b/CD18) and Fc gamma RIII cooperate in generation of a neutrophil respiratory burst: requirement for Fc gamma RIII and tyrosine phosphorylation. J Cell Biol. (1994) 125:1407–16. 10.1083/jcb.125.6.1407 7515890 PMC2290913

[171] Salmon JE Edberg JC Kimberly RP. Fc gamma receptor III on human neutrophils. Allelic variants have functionally distinct capacities. J Clin Invest. (1990) 85:1287–95. 10.1172/JCI114566 1690757 PMC296565

[172] Koene HR Kleijer M Roos D de Haas M Von dem Borne AE. Fc gamma RIIIB gene duplication: evidence for presence and expression of three distinct Fc gamma RIIIB genes in NA(1+,2+)SH(+) individuals. Blood. (1998) 91:673–9. 9427724

[173] Carter NA Harnett MM Dissection of the signalling mechanisms underlying Fcγ RIIB-mediated apoptosis of mature B-cells. Biochem Soc Trans. (2004) 32(Pt 6):973-975. 10.1042/BST0320973 15506939

[174] Xiang Z Cutler AJ Brownlie RJ Fairfax K Lawlor KE Severinson E. FcgammaRIIb controls bone marrow plasma cell persistence and apoptosis. Nat Immunol. (2007) 8:419–29. 10.1038/ni1440 17322888

[175] McKenzie ANJ Spits H Eberl G. Innate lymphoid cells in inflammation and immunity. Immunity. (2014) 41:366–74. 10.1016/j.immuni.2014.09.006 25238094

[176] Artis D Spits H. The biology of innate lymphoid cells. Nature. 517:293–301. 10.1038/nature14189 25592534

[177] Klose CSN Kiss EA Schwierzeck V Ebert K Hoyler T d'Hargues Y. A T-bet gradient controls the fate and function of CCR6-RORγt+ innate lymphoid cells. Nature. (2013) 494:261–5. 10.1038/nature11813 23334414

[178] Lee JS Cella M McDonald KG Garlanda C Kennedy GD Nukaya M. AHR drives the development of gut ILC22 cells and postnatal lymphoid tissues via pathways dependent on and independent of Notch. Nat Immunol. (2012) 13:144–51. 10.1038/ni.2187 22101730 PMC3468413

[179] Sanos SL Bui VL Mortha A Oberle K Heners C Johner C. RORgammat and commensal microflora are required for the differentiation of mucosal interleukin 22-producing NKp46+ cells. Nat Immunol. (2009) 10:83–91. 10.1038/ni.1684 19029903 PMC4217274

[180] Zook EC Kee BL. Development of innate lymphoid cells. Nat Immunol. (2016) 17:775–775. 10.1038/ni.3481 27328007

[181] Eberl G Marmon S Sunshine M-J Rennert PD Choi Y Littman DR. An essential function for the nuclear receptor RORgamma(t) in the generation of fetal lymphoid tissue inducer cells. Nat Immunol. (2004) 5:64–73. 10.1038/ni1022 14691482

[182] Hepworth MR Fung TC Masur SH Kelsen JR McConnell FM Dubrot J. Group 3 innate lymphoid cells mediate intestinal selection of commensal bacteria-specific CD4+ T cells. Science. (2015) 348:1031–5. 10.1126/science.aaa4812 25908663 PMC4449822

[183] Hepworth MR Monticelli LA Fung TC Ziegler CGK Grunberg S Sinha R. Innate lymphoid cells regulate CD4+ T-cell responses to intestinal commensal bacteria. Nature. (2013) 498:113–7. 10.1038/nature12240 23698371 PMC3699860

[184] Takatori H Kanno Y Watford WT Tato CM Weiss G Ivanov II. Lymphoid tissue inducer-like cells are an innate source of IL-17 and IL-22. J Exp Med. (2009) 206:35–41. 10.1084/jem.20072713 19114665 PMC2626689

[185] Zheng Y Valdez PA Danilenko DM Hu Y Sa SM Gong Q. Interleukin-22 mediates early host defense against attaching and effacing bacterial pathogens. Nat Med. (2008) 14:282–9. 10.1038/nm1720 18264109

[186] Goto Y Obata T Kunisawa J Sato S Ivanov II Lamichhane A. Innate lymphoid cells regulate intestinal epithelial cell glycosylation. Science. (2014) 345:1254009. 10.1126/science.1254009 25214634 PMC4774895

[187] Hanash AM Dudakov JA Hua G O'Connor MH Young LF Singer NV. Interleukin-22 protects intestinal stem cells from immune-mediated tissue damage and regulates sensitivity to graft versus host disease. Immunity. (2012) 37:339–50. 10.1016/j.immuni.2012.05.028 22921121 PMC3477611

[188] Lindemans CA Calafiore M Mertelsmann AM O'Connor MH Dudakov JA Jenq RR. Interleukin-22 promotes intestinal-stem-cell-mediated epithelial regeneration. Nature. (2015) 528:560–4. 10.1038/nature16460 26649819 PMC4720437

[189] Pham TAN Clare S Goulding D Arasteh JM Stares MD Browne HP. Epithelial IL-22RA1-mediated fucosylation promotes intestinal colonization resistance to an opportunistic pathogen. Cell Host and Microbe. (2014) 16:504–16. 10.1016/j.chom.2014.08.017 25263220 PMC4190086

[190] Pickert G Neufert C Leppkes M Zheng Y Wittkopf N Warntjen M. STAT3 links IL-22 signaling in intestinal epithelial cells to mucosal wound healing. J Exp Med. (2009) 206:1465–72. 10.1084/jem.20082683 19564350 PMC2715097

[191] Sonnenberg GF Monticelli LA Alenghat T Fung TC Hutnick Na Kunisawa J. Innate lymphoid cells promote anatomical containment of lymphoid-resident commensal bacteria. Science. (2012) 336:1321–5. 10.1126/science.1222551 22674331 PMC3659421

[192] Griseri T McKenzie BS Schiering C Powrie F. Dysregulated hematopoietic stem and progenitor cell activity promotes interleukin-23-driven chronic intestinal inflammation. Immunity. (2012) 37:1116–29. 10.1016/j.immuni.2012.08.025 23200826 PMC3664922

[193] Pearson C Thornton EE McKenzie B Schaupp A-L Huskens N Griseri T. ILC3 GM-CSF production and mobilisation orchestrate acute intestinal inflammation. eLife. (2016) 5:e10066. 10.7554/eLife.10066 26780670 PMC4733039

[194] Song C Lee JS Gilfillan S Robinette ML Newberry RD Stappenbeck TS. Unique and redundant functions of NKp46+ ILC3s in models of intestinal inflammation. J Exp Med. (2015) 212:1869–82. 10.1084/jem.20151403 26458769 PMC4612098

[195] Metes D Ernst LK Chambers WH Sulica A Herberman RB Morel PA. Expression of functional CD32 molecules on human NK cells is determined by an allelic polymorphism of the FcgammaRIIC gene. Blood. (1998) 91:2369–80. 9516136

[196] Vivier E Tomasello E Baratin M Walzer T Ugolini S. Functions of natural killer cells. Nat Immunol. (2008) 9:503–10. 10.1038/ni1582 18425107

[197] Bryceson YT March ME Ljunggren H-G Long EO. Synergy among receptors on resting NK cells for the activation of natural cytotoxicity and cytokine secretion. Blood. (2006) 107:159–67. 10.1182/blood-2005-04-1351 16150947 PMC1895346

[198] Srivastava S Pelloso D Feng H Voiles L Lewis D Haskova Z. Effects of interleukin-18 on natural killer cells: costimulation of activation through Fc receptors for immunoglobulin. Cancer Immunol Immunother. (2013) 62:1073–82. 10.1007/s00262-013-1403-0 23604103 PMC3707624

[199] Prehn JL Thomas LS Landers CJ Yu QT Michelsen KS Targan SR. The T cell costimulator TL1A is induced by FcgammaR signaling in human monocytes and dendritic cells. J Immunol. (2007) 178:4033–8. 10.4049/jimmunol.178.7.4033 17371957

[200] Uo M Hisamatsu T Miyoshi J Kaito D Yoneno K Kitazume MT. Mucosal CXCR4+ IgG plasma cells contribute to the pathogenesis of human ulcerative colitis through FcγR-mediated CD14 macrophage activation. Gut. (2013) 62:1734–44. 10.1136/gutjnl-2012-303063 23013725

[201] Allan DS Kirkham CL Aguilar OA Qu LC Chen P Fine JH. An *in vitro* model of innate lymphoid cell function and differentiation. Mucosal Immunol. (2014) 8: 340–51. 10.1038/mi.2014.71 25138665

[202] Koues OI Collins PL Cella M Robinette ML Porter SI Pyfrom SC. Distinct gene regulatory pathways for human innate versus adaptive lymphoid cells. Cell. (2016) 165:1–13. 10.1016/j.cell.2016.04.014 27156452 PMC4874868

[203] Renz H Brandtzaeg P Hornef M. The impact of perinatal immune development on mucosal homeostasis and chronic inflammation. Nat Rev Immunol. (2012) 12:9–23. 10.1038/nri3112 22158411

[204] Slack E Hapfelmeier S Stecher B Velykoredko Y Stoel M Lawson MAE. Innate and adaptive immunity cooperate flexibly to maintain host-microbiota mutualism. Science. (2009) 325:617–20. 10.1126/science.1172747 19644121 PMC3730530

[205] Zeng MY Cisalpino D Varadarajan S Hellman J Warren HS Cascalho M. Gut microbiota-induced immunoglobulin G controls systemic infection by symbiotic bacteria and pathogens. Immunity. (2016) 44:647–58. 10.1016/j.immuni.2016.02.006 26944199 PMC4794373

[206] Harris NL Spoerri I Schopfer JF Nembrini C Merky P Massacand J. Mechanisms of neonatal mucosal antibody protection. J Immunol. (2006) 177:6256–62. 10.4049/jimmunol.177.9.6256 17056555

[207] Qiu J Heller JJ Guo X Chen Z-ME Fish K Fu Y-X. The aryl hydrocarbon receptor regulates gut immunity through modulation of innate lymphoid cells. Immunity. (2012) 36:92–104. 10.1016/j.immuni.2011.11.011 22177117 PMC3268875

[208] Bruhns P Jönsson F. Mouse and human FcR effector functions. Immunol Rev. (2015) 268:25–51. 10.1111/imr.12350 26497511

[209] Moor K Fadlallah J Toska A Sterlin D Balmer ML Macpherson AJ. Analysis of bacterial-surface-specific antibodies in body fluids using bacterial flow cytometry. Nat Protoc. (2016) 11:1531–53. 10.1038/nprot.2016.091 27466712

[210] Bry L Brenner MB Critical role of T cell-dependent serum antibody, but not the gut-associated lymphoid tissue, for surviving acute mucosal infection with Citrobacter rodentium, an attaching and effacing pathogen. J Immunol. (2004) 172:433–41. 10.4049/jimmunol.172.1.433 14688352

[211] Simmons CP Clare S Ghaem-Maghami M Uren TK Rankin J Huett A. Central role for B lymphocytes and CD4+ T cells in immunity to infection by the attaching and effacing pathogen *Citrobacter rodentium*. Infect Immun. (2003) 71:5077–86. 10.1128/IAI.71.9.5077-5086.2003 12933850 PMC187366

[212] Kim YG Kamada N Shaw MH Warner N Chen GY Franchi L. The Nod2 sensor promotes intestinal pathogen eradication via the chemokine CCL2-dependent recruitment of inflammatory monocytes. Immunity. (2011) 34:769–80. 10.1016/j.immuni.2011.04.013 21565531 PMC3103637

[213] Schreiber HA Loschko J Karssemeijer RA Escolano A Meredith MM Mucida D. Intestinal monocytes and macrophages are required for T cell polarization in response to Citrobacter rodentium. J Exp Med. (2013) 210:2025–39. 10.1084/jem.20130903 24043764 PMC3782042

[214] Mohr E Cunningham AF Toellner K-M Bobat S Coughlan RE Bird RA. IFN-{gamma} produced by CD8 T cells induces T-bet-dependent and -independent class switching in B cells in responses to alum-precipitated protein vaccine. Proc Natl Acad Sci USA. (2010) 107:17292–7. 10.1073/pnas.1004879107 20855629 PMC2951392

[215] Peng SL Szabo SJ Glimcher LH. T-bet regulates IgG class switching and pathogenic autoantibody production. Proc Natl Acad Sci USA. (2002) 99:5545–50. 10.1073/pnas.082114899 11960012 PMC122806

[216] Belzer C Liu Q Carroll MC Bry L. The role of specific IgG and complement in combating a primary mucosal infection of the gut epithelium. Eur J Microbiol Immunol. (2011) 1:311–8. 10.1556/EuJMI.1.2011.4.7 22485193 PMC3319158

[217] Masuda A Yoshida M Shiomi H Ikezawa S Takagawa T Tanaka H. Fcgamma receptor regulation of Citrobacter rodentium infection. Infect Immun. (2008) 76:1728–37. 10.1128/IAI.01493-07 18227164 PMC2292883

[218] Caballero-Flores G Sakamoto K Zeng MY Wang Y Hakim J Matus-Acuña V. Maternal immunization confers protection to the offspring against an attaching and effacing pathogen through delivery of IgG in breast milk. Cell Host Microbe. (2019) 25:313–23. 10.1016/j.chom.2018.12.015 30686564 PMC6375740

[219] Ben Suleiman Y Yoshida M Nishiumi S Tanaka H Mimura T Nobutani K Neonatal Fc receptor for IgG (FcRn) expressed in the gastric epithelium regulates bacterial infection in mice. Mucosal Immunol. (2012) 5:87–98. 10.1038/mi.2011.53 22089027 PMC3964614

[220] Baba TW Liska V Hofmann-Lehmann R Vlasak J Xu W Ayehunie S. Human neutralizing monoclonal antibodies of the IgG1 subtype protect against mucosal simian-human immunodeficiency virus infection. Nat Med. (2000) 6:200–6. 10.1038/72309 10655110

[221] Mascola JR Stiegler G VanCott TC Katinger H Carpenter CB Hanson CE. Protection of macaques against vaginal transmission of a pathogenic HIV-1/SIV chimeric virus by passive infusion of neutralizing antibodies. Nat Med. (2000) 6:207–10. 10.1038/72318 10655111

[222] Bournazos S Klein F Pietzsch J Seaman MS Nussenzweig MC Ravetch JV. Broadly neutralizing anti-HIV-1 antibodies require Fc effector functions for *in vivo* activity. Cell. (2014) 158:1243–53. 10.1016/j.cell.2014.08.023 25215485 PMC4167398

[223] Ko S-Y Pegu A Rudicell RS Yang Z-Y Joyce MG Chen X. Enhanced neonatal Fc receptor function improves protection against primate SHIV infection. Nature. (2014) 514:642–5. 10.1038/nature13612 25119033 PMC4433741

[224] Ackerman ME Das J Pittala S Broge T Linde C Suscovich TJ. Route of immunization defines multiple mechanisms of vaccine-mediated protection against SIV. Nat Med. (2018) 24:1590–8. 10.1038/s41591-018-0161-0 30177821 PMC6482471

[225] Westerman LE McClure HM Jiang B Almond JW Glass RI. Serum IgG mediates mucosal immunity against rotavirus infection. Proc Natl Acad Sci USA. (2005) 102:7268–73. 10.1073/pnas.0502437102 15883382 PMC1129131

[226] Cho JH. The genetics and immunopathogenesis of inflammatory bowel disease. Nat Rev Immunol. (2008) 8:458–66. 10.1038/nri2340 18500230

[227] Xavier RJ Podolsky DK. Unravelling the pathogenesis of inflammatory bowel disease. Nature. (2007) 448:427–34. 10.1038/nature06005 17653185

[228] Burton PR Clayton DG Cardon LR Craddock N Deloukas P Duncanson A. Association scan of 14,500 nonsynonymous SNPs in four diseases identifies autoimmunity variants. Nat Genet. (2007) 39:1329–37. 10.1038/ng.2007.17 17952073 PMC2680141

[229] Elson CO Cong Y Weaver CT Schoeb TR McClanahan TK Fick RB. Monoclonal anti-interleukin 23 reverses active colitis in a T cell-mediated model in mice. Gastroenterology. (2007) 132:2359–70. 10.1053/j.gastro.2007.03.104 17570211

[230] Hue S Ahern P Buonocore S Kullberg MC Cua DJ McKenzie BS. Interleukin-23 drives innate and T cell-mediated intestinal inflammation. J Exp Med. (2006) 203:2473–83. 10.1084/jem.20061099 17030949 PMC2118132

[231] Kullberg MC Jankovic D Feng CG Hue S Gorelick PL McKenzie BS. IL-23 plays a key role in Helicobacter hepaticus-induced T cell-dependent colitis. J Exp Med. (2006) 203:2485–94. 10.1084/jem.20061082 17030948 PMC2118119

[232] Van den Brande JMH Braat H Van den Brink GR Versteeg HH Bauer CA Hoedemaeker I Infliximab but not etanercept induces apoptosis in lamina propria T-lymphocytes from patients with Crohn's disease. Gastroenterology. (2003) 124:1774–85. 10.1016/S0016-5085(03)00382-2 12806611

[233] Mannon PJ Fuss IJ Mayer L Elson CO Sandborn WJ Present D. Anti-interleukin-12 antibody for active Crohn's disease. N Engl J Med. (2004) 351:2069–79. 10.1056/NEJMoa033402 15537905

[234] Sandborn WJ Gasink C Gao L-L Blank MA Johanns J Guzzo C. Ustekinumab induction and maintenance therapy in refractory Crohn's disease. N Engl J Med. (2012) 367:1519–28. 10.1056/NEJMoa1203572 23075178

[235] Kolaczkowska E Kubes P. Neutrophil recruitment and function in health and inflammation. Nat Rev Immunol. (2013) 13:159–75. 10.1038/nri3399 23435331

[236] Chung Y Chang SH Martinez GJ Yang XO Nurieva R Kang HS. Critical regulation of early Th17 cell differentiation by Interleukin-1 signaling. Immunity. (2009) 30:576–87. 10.1016/j.immuni.2009.02.007 19362022 PMC2705871

[237] Ghoreschi K Laurence A Yang X-P Tato CM Mandy JM Konkel J Generation of pathogenic Th17 cells in the absence of TGF-B signaling. Nature. (2010) 467:967–71. 10.1038/nature09447 20962846 PMC3108066

[238] Revu S Wu J Henkel M Rittenhouse N Menk A Delgoffe GM. IL-23 and IL-1β drive human Th17 cell differentiation and metabolic reprogramming in absence of CD28 costimulation. Cell Rep. (2018) 22:2642–53. 10.1016/j.celrep.2018.02.044 29514093 PMC5884137

[239] Macpherson A Khoo UY Forgacs I Philpott-Howard J Bjarnason I. Mucosal antibodies in inflammatory bowel disease are directed against intestinal bacteria. Gut. (1996) 38:365–75. 10.1136/gut.38.3.365 8675088 PMC1383064

[240] Kuna AT. Serological markers of inflammatory bowel disease. Biochem Med. (2013) 23:28–42. 10.11613/BM.2013.006 23457764 PMC3900099

[241] Quinton JF Sendid B Reumaux D Duthilleul P Cortot A Grandbastien B. Anti-Saccharomyces cerevisiae mannan antibodies combined with antineutrophil cytoplasmic autoantibodies in inflammatory bowel disease: prevalence and diagnostic role. Gut. (1998) 42:788–91. 10.1136/gut.42.6.788 9691915 PMC1727156

[242] Teegen B Niemann S Probst C Schlumberger W Stöcker W Komorowski L. DNA-bound lactoferrin is the major target for antineutrophil perinuclear cytoplasmic antibodies in ulcerative colitis. Ann N Y Acad Sci. (2009) 1173:161–5. 10.1111/j.1749-6632.2009.04752.x 19758145

[243] Hibi T Ohara M Kobayashi K Brown WR Toda K Takaishi H. Enzyme linked immunosorbent assay (ELISA) and immunoprecipitation studies on anti-goblet cell antibody using a mucin producing cell line in patients with inflammatory bowel disease. Gut. (1994) 35:224–30. 10.1136/gut.35.2.224 8307474 PMC1374498

[244] Gillis C Gouel-Chéron A Jönsson F Bruhns P. Contribution of human FcγRs to disease with evidence from human polymorphisms and transgenic animal studies. Frontiers in Immunology. (2014) 5:1–13. 10.3389/fimmu.2014.00254 24910634 PMC4038777

[245] Warmerdam PA van de Winkel JG Gosselin EJ Capel PJ. Molecular basis for a polymorphism of human Fc gamma receptor II (CD32). J Exp Med. (1990) 172:19–25. 10.1084/jem.172.1.19 2141627 PMC2188138

[246] van der Heijden J Nagelkerke S Zhao X Geissler J Rispens T van den Berg TK Haplotypes of FcgammaRIIa and FcgammaRIIIb polymorphic variants influence IgG-mediated responses in neutrophils. J Immunol. (2014) 192:2715–21. 10.4049/jimmunol.1203570 24554771

[247] Chuen Khor C Davila S Breunis WB Lee Y-C Shimizu C Wright VJ Genome-wide association study identifies FCGR2A as a susceptibility locus for Kawasaki disease. Nat Genet. (2011) 43:1241–6. 10.1038/ng.981 22081228

[248] Castro-Dopico T Dennison TW Ferdinand JR Mathews RJ Fleming A Clift D. Anti-commensal IgG drives intestinal inflammation and type 17 immunity in ulcerative colitis. Immunity. 10.1016/j.immuni.2019.02.006. [Epub ahead of print]. 30876876 PMC6477154

[249] Kobayashi K Qiao SW Yoshida M Baker K Lencer WI Blumberg RS. An FcRn-Dependent role for anti-flagellin immunoglobulin G in pathogenesis of colitis in mice. Gastroenterology. (2009) 137:174656.e1741. 10.1053/j.gastro.2009.07.059 19664634 PMC2787451

[250] Floto RA Clatworthy MR Heilbronn KR Rosner DR MacAry PA Rankin A. Loss of function of a lupus-associated FcgammaRIIb polymorphism through exclusion from lipid rafts. Nat Med. (2005) 11:1056–8. 10.1038/nm1288 16170323

[251] Kono H Kyogoku C Suzuki T Tsuchiya N Honda H Yamamoto K. FcgammaRIIB Ile232Thr transmembrane polymorphism associated with human systemic lupus erythematosus decreases affinity to lipid rafts and attenuates inhibitory effects on B cell receptor signaling. Hum Mol Genet. (2005) 14:2881–92. 10.1093/hmg/ddi320 16115811

[252] Xu L Xia M Guo J Sun X Li H Xu C Impairment on the lateral mobility induced by structural changes underlies the functional deficiency of the lupus- associated polymorphism Fc γ RIIB-T232. J Exp Med. (2016) 213:2707–27. 10.1084/jem.20160528 27799621 PMC5110019

[253] Kyogoku C Dijstelbloem HM Tsuchiya N Hatta Y Kato H Yamaguchi A. Fcgamma receptor gene polymorphisms in Japanese patients with systemic lupus erythematosus: contribution of FCGR2B to genetic susceptibility. Arthritis Rheum. (2002) 46:1242–54. 10.1002/art.10257 12115230

[254] Willcocks LC Carr EJ Niederer HA Rayner TF Williams TN Yang W. A defunctioning polymorphism in FCGR2B is associated with protection against malaria but susceptibility to systemic lupus erythematosus. Proc Natl Acad Sci USA. (2010) 107:7881–5. 10.1073/pnas.0915133107 20385827 PMC2867866

[255] Clatworthy MR Willcocks L Urban B Langhorne J Williams TN Peshu N. Systemic lupus erythematosus-associated defects in the inhibitory receptor FcgammaRIIb reduce susceptibility to malaria. Proc Natl Acad Sci USA. (2007) 104:7169–74. 10.1073/pnas.0608889104 17435165 PMC1855357

[256] Morgan AW Griffiths B Ponchel F Montague BM Ali M Gardner PP. Fcgamma receptor type IIIA is associated with rheumatoid arthritis in two distinct ethnic groups. Arthritis Rheum. (2000) 43:2328–34. 10.1002/1529-0131(200010)43:10<2328::AID-ANR21>3.0.CO;2-Z 11037893

[257] Tutuncu Z Kavanaugh A Zvaifler N Corr M Deutsch R Boyle D. Fcgamma receptor type IIIA polymorphisms influence treatment outcomes in patients with inflammatory arthritis treated with tumor necrosis factor alpha-blocking agents. Arthritis Rheum. (2005) 52:2693–6. 10.1002/art.21266 16142749

[258] Niederer HA Willcocks LC Rayner TF Yang W Lau YL Williams TN. Copy number, linkage disequilibrium and disease association in the FCGR locus. Hum Mol Genet. (2010) 19:3282–94. 10.1093/hmg/ddq216 20508037 PMC2908468

[259] Chung AW Kumar MP Arnold KB Yu WH Schoen MK Dunphy LJ. Dissecting polyclonal vaccine-induced humoral immunity against HIV using systems serology. Cell. (2015) 163:988–98. 10.1016/j.cell.2015.10.027 26544943 PMC5490491

[260] Lofano G Gorman MJ Yousif AS Yu W-H Fox JM Dugast A-S. Antigen-specific antibody Fc glycosylation enhances humoral immunity via the recruitment of complement. Sci Immunol. (2018) 3:eaat7796. 10.1126/sciimmunol.aat7796 30120121 PMC6298214

[261] Lu LL Chung AW Rosebrock TR Ghebremichael M Yu WH Grace PS. A functional role for antibodies in tuberculosis. Cell. (2016) 167:433–43.e414. 10.1016/j.cell.2016.08.072 27667685 PMC5526202

[262] Bernard NJ. Rheumatoid arthritis: changes in ACPA Fc glycosylation patterns prior to RA onset. Nat Rev Rheum. (2013) 9:697–697. 10.1038/nrrheum.2013.162 24166244

[263] Vučkovïc F Krištïc J Gudelj I Teruel M Keser T Pezer M. Association of systemic lupus erythematosus with decreased immunosuppressive potential of the IgG glycome. Arthritis Rheumatol. (2015) 67:2978–89. 10.1002/art.39273 26200652 PMC4626261

[264] Miyoshi E Shinzaki S Fujii H Iijima H Kamada Y Takehara T. Role of aberrant IgG glycosylation in the pathogenesis of inflammatory bowel disease. Proteomics. (2016) 10:384–90. 10.1002/prca.201500089 26427763

[265] Trbojević Akmačić I Ventham NT Theodoratou E Vučković F Kennedy NA Krištić J Inflammatory bowel disease associates with proinflammatory potential of the immunoglobulin G glycome. Inflamm Bowel Dis. (2015) 21:1 10.1097/MIB.0000000000000372 25895110 PMC4450892

[266] Quast I Keller CW Maurer MA Giddens JP Tackenberg B Wang LX. Sialylation of IgG Fc domain impairs complement-dependent cytotoxicity. J Clin Invest. (2015) 125:4160–70. 10.1172/JCI82695 26436649 PMC4639970

[267] Pfeifle R Rothe T Ipseiz N Scherer HU Culemann S Harre U Regulation of autoantibody activity by the IL-23 – T H 17 axis determines the onset of autoimmune disease. Nat Immunol. (2016) 18:104–13. 10.1038/ni.3579 27820809 PMC5164937

[268] Baker K Rath T Flak MB Arthur JC Chen Z Glickman JN. Neonatal Fc receptor expression in dendritic cells mediates protective immunity against Colorectal Cancer. Immunity. (2013) 39:1095–107. 10.1016/j.immuni.2013.11.003 24290911 PMC3902970

[269] Asano K Matsumoto T Umeno J Hirano A Esaki M Hosono N. Impact of allele copy number of polymorphisms in FCGR3A and FCGR3B genes on susceptibility to ulcerative colitis. Inflamm Bowel Dis. (2013) 19:2061–8. 10.1097/MIB.0b013e318298118e 23917248

[270] Carmi Y Spitzer MH Linde IL Burt BM Prestwood TR Perlman N. Allogeneic IgG combined with dendritic cell stimuli induce antitumour T-cell immunity. Nature. (2015) 521:99–104. 10.1038/nature14424 25924063 PMC4877172

[271] Ferrari de Andrade L Tay RE Pan D Luoma AM Ito Y Badrinath S Antibody-mediated inhibition of MICA and MICB shedding promotes NK cell–driven tumor immunity. Science. (2018) 359:1537–42. 10.1126/science.aao0505 29599246 PMC6626532

[272] Rogosnitzky M Danks R Holt D. Intravenous immunoglobulin for the treatment of Crohn's disease. Autoimmun Rev. (2012) 12:275–80. 10.1016/j.autrev.2012.04.006 22579561

[273] Shah S Terdiman J Gundling K Mahadevan U. Immunoglobulin therapy for refractory Crohn's disease. Ther Adv Gastroenterol. (2013) 7:99–102. 10.1177/1756283X13504728 24587823 PMC3903086

[274] Horton N Kochhar G Patel K Lopez R Shen B Efficacy and factors associated with treatment response of intravenous immunoglobulin in inpatients with refractory *Inflamm Bowel Dis*. (2017) 23:1080–7. 10.1097/MIB.0000000000001116 28452863

[275] Leiper K Martin K Ellis A Subramanian S Watson AJ Christmas SE. Randomised placebo-controlled trial of rituximab (anti-CD20) in active ulcerative colitis. Gut. (2011) 60:1520–6. 10.1136/gut.2010.225482 21471566

[276] Clatworthy MR Watson CJE Plotnek G Bardsley V Chaudhry AN Bradley JA. B-cell–depleting induction therapy and acute cellular rejection. N Engl J Med. (2009) 360:2683–5. 10.1056/NEJMc0808481 19535812 PMC4143588

[277] Flores-Borja F Bosma A Ng D Reddy V Ehrenstein MR Isenberg DA. CD19+CD24hiCD38hi B cells maintain regulatory T cells while limiting TH1 and TH17 differentiation. Sci Transl Med. (2013) 5:173ra123. 10.1126/scitranslmed.3005407 23427243

[278] Menon M Blair PA Isenberg DA Mauri C. A regulatory feedback between plasmacytoid dendritic cells and regulatory B cells is aberrant in systemic lupus erythematosus. Immunity. (2016) 44:683–97. 10.1016/j.immuni.2016.02.012 26968426 PMC4803914

[279] Edwards JCW Szczepanski L Szechinski J Filipowicz-Sosnowska A Emery P Close DR. Efficacy of B-cell-targeted therapy with rituximab in patients with rheumatoid arthritis. N Engl J Med. (2004) 350:2572–81. 10.1056/NEJMoa032534 15201414

[280] Kiessling P Lledo-Garcia R Watanabe S Langdon G Tran D Bari M. The FcRn inhibitor rozanolixizumab reduces human serum IgG concentration: a randomized phase 1 study. Sci Transl Med. (2017) 9:1–13. 10.1126/scitranslmed.aan1208 29093180

[281] Ling L Hillson JL Tiessen RG Bosje T van Iersel MP Nix DJ. M281, an Anti-FcRn antibody: pharmacodynamics, pharmacokinetics, and safety across the full range of IgG reduction in a first-in-human study. Clin Pharm Ther. (2018) 105:1031–9. 10.1002/cpt.1276 30402880 PMC6587432

[282] Smith B Kiessling A Lledo-Garcia R Dixon KL Christodoulou L Catley MC. Generation and characterization of a high affinity anti-human FcRn antibody, rozanolixizumab, and the effects of different molecular formats on the reduction of plasma IgG concentration. MABS. (2018) 10:1111–30. 10.1080/19420862.2018.1505464 30130439 PMC6291300

[283] Ulrichts P Guglietta A Dreier T van Bragt T Hanssens V Hofman E. Neonatal Fc receptor antagonist efgartigimod safely and sustainably reduces IgGs in humans. J Clin Invest. (2018) 128:4372–86. 10.1172/JCI97911 30040076 PMC6159959

[284] Weinblatt ME Kavanaugh A Genovese MC Musser TK Grossbard EB Magilavy DB. An oral spleen tyrosine kinase (Syk) inhibitor for rheumatoid arthritis. N Engl J Med. (2010) 363:1303–12. 10.1056/NEJMoa1000500 20879879

[285] Bosques CJ Manning AM. Fc-gamma receptors: attractive targets for autoimmune drug discovery searching for intelligent therapeutic designs. Autoimmun Rev. (2016) 15:1081–8. 10.1016/j.autrev.2016.07.035 27491569

[286] Horton HM Chu SY Ortiz EC Pong E Cemerski S Leung IWL. Antibody-mediated coengagement of FcγRIIb and B cell receptor complex suppresses humoral immunity in systemic lupus erythematosus. J Immunol. (2011) 186:4223–33. 10.4049/jimmunol.1003412 21357255

[287] Louis E El Ghoul Z Vermeire S Dall'Ozzo S Rutgeerts P Paintaud G. Association between polymorphism in IgG Fc receptor IIIa coding gene and biological response to infliximab in Crohn's disease. Aliment Pharmacol Ther. (2004) 19:511–9. 10.1111/j.1365-2036.2004.01871.x 14987319

[288] Moroi R Endo K Kinouchi Y Shiga H Kakuta Y Kuroha M. FCGR3A-158 polymorphism influences the biological response to infliximab in Crohn's disease through affecting the ADCC activity. Immunogenetics. (2013) 65:265–71. 10.1007/s00251-013-0679-8 23358932

[289] Bloemendaal FM Koelink PJ van Schie KA Rispens T Peters CP Buskens CJ TNF-anti-TNF immune complexes inhibit IL-12/IL-23 secretion by inflammatory macrophages via an Fc-dependent mechanism. J Crohns Colitis. (2018) 12:1122–30. 10.1093/ecco-jcc/jjy075 29860435

[290] McRae BL Levin AD Wildenberg ME Koelink PJ Bousquet P Mikaelian I. Fc receptor-mediated effector function contributes to the therapeutic response of anti-TNF monoclonal antibodies in a mouse model of inflammatory bowel disease. J Crohns Colitis. (2016) 10:69–76. 10.1093/ecco-jcc/jjv179 26429698

[291] Weng WK Levy R. Two immunoglobulin G fragment C receptor polymorphisms independently predict response to rituximab in patients with follicular lymphoma. J Clin Oncol. (2003) 21:3940–7. 10.1200/JCO.2003.05.013 12975461

[292] Arce Vargas F Furness AJS Solomon I Joshi K Mekkaoui L Lesko MH Fc-optimized anti-CD25 depletes tumor-infiltrating regulatory T cells and synergizes with PD-1 blockade to eradicate established tumors. Immunity. (2017) 46:577–86. 10.1016/j.immuni.2017.03.013 28410988 PMC5437702

[293] Vargas FA Furness AJS Litchfield K Joshi K Rosenthal R Ghorani E Fc effector function contributes to the activity of human anti-CTLA-4 antibodies. Cancer Cell. (2018) 33:649–63.e644. 10.1016/j.ccell.2018.02.010 29576375 PMC5904288

[294] Ingram JR Blomberg OS Rashidian M Ali L Garforth S Fedorov E. Anti – CTLA-4 therapy requires an Fc domain for efficacy. Proc Natl Acad Sci USA. (2018) 115:3912–7. 10.1073/pnas.1801524115 29581255 PMC5899492

[295] Waight JD Chand D Dietrich S Gombos R Horn T Gonzalez AM. Selective FcγR Co-engagement on APCs modulates the activity of therapeutic antibodies targeting T cell antigens. Cancer Cell. (2018) 33:1033–1047.e1035. 10.1016/j.ccell.2018.05.005 29894690 PMC6292441

[296] Liu G Tu D Lewis M Cheng D Sullivan LA Chen Z Fc-g receptor polymorphisms, cetuximab therapy, and survival in the NCIC CTG CO.17 trial of colorectal cancer. Clin Cancer Res. (2016) 22:2435–44. 10.1158/1078-0432.CCR-15-0414 27179112

[297] Weersma RK Crusius JB Roberts RL Koeleman BP Palomino-Morales R Wolfkamp S Association of FcgR2a, but not FcgR3a, with inflammatory bowel diseases across three Caucasian populations. Inflamm Bowel Dis. (2010) 16:2080–9. 10.1002/ibd.21342 20848524

[298] Yang SK Jung Y Kim H Hong M Ye BD Song K. Association of FCGR2A, JAK2 or HNF4A variants with ulcerative colitis in Koreans. Dig Liver Dis. (2011) 43:856–61. 10.1016/j.dld.2011.07.006 21831733

